# Site-Specific ^68^Ga Radiolabeling of Trastuzumab Fab via Methionine for ImmunoPET Imaging

**DOI:** 10.1021/acs.bioconjchem.3c00344

**Published:** 2023-09-26

**Authors:** Thomas T. C. Yue, Ying Ge, Francesco A. Aprile, Michelle T. Ma, Truc T. Pham, Nicholas J. Long

**Affiliations:** † Department of Chemistry, Molecular Sciences Research Hub, Imperial College London, White City Campus, Wood Lane, London W120BZ, U.K.; ‡ School of Biomedical Engineering and Imaging Sciences, King’s College London, Fourth Floor Lambeth Wing, St. Thomas’ Hospital, London SE17EH, U.K.; § Department of Chemistry and Institute of Chemical Biology, Molecular Sciences Research Hub, Imperial College London, White City Campus, Wood Lane, London W120BZ, U.K.

## Abstract

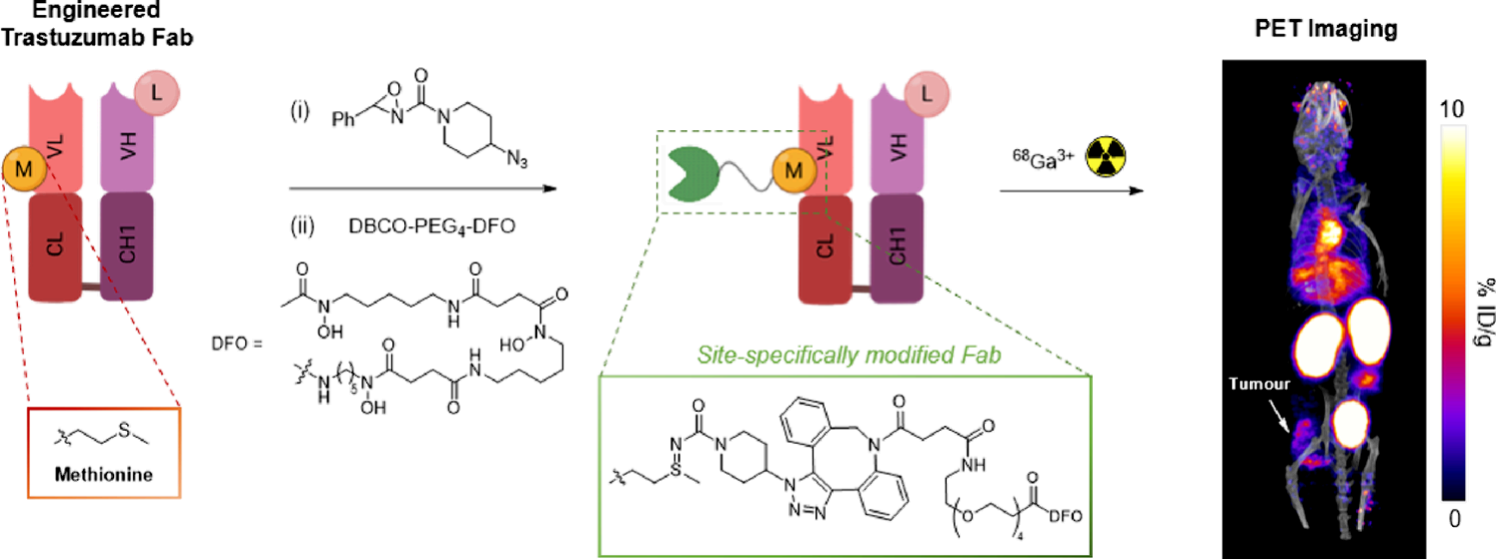

Bioconjugates of antibodies and their derivatives radiolabeled with β^+^-emitting radionuclides can be utilized for diagnostic PET imaging. Site-specific attachment of radioactive cargo to antibody delivery vectors provides homogeneous, well-defined immunoconjugates. Recent studies have demonstrated the utility of oxaziridine chemistry for site-specific labeling of methionine residues. Herein, we applied this approach to site-specifically radiolabel trastuzumab-derived Fab immunoconjugates with ^68^Ga, which can be used for in vivo PET imaging of HER2-positive breast cancer tumors. Initially, a reactive azide was introduced to a single solvent-accessible methionine residue in both the wild-type Fab and an engineered derivative containing methionine residue M74, utilizing the principles of oxaziridine chemistry. Subsequently, these conjugates were functionalized with a modified DFO chelator incorporating dibenzocyclooctyne. The resulting DFO-WT and DFO-M74 conjugates were radiolabeled with generator-produced [^68^Ga]Ga^3+^, to yield the novel PET radiotracers, [^68^Ga]Ga-DFO-WT and [^68^Ga]Ga-DFO-M74. In vitro and in vivo studies demonstrated that [^68^Ga]Ga-DFO-M74 exhibited a higher affinity for HER2 receptors. Biodistribution studies in mice bearing orthotopic HER2-positive breast tumors revealed a higher uptake of [^68^Ga]Ga-DFO-M74 in the tumor tissue, accompanied by rapid renal clearance, enabling clear delineation of tumors using PET imaging. Conversely, [^68^Ga]Ga-DFO-WT exhibited lower uptake and inferior image contrast compared to [^68^Ga]Ga-DFO-M74. Overall, the results demonstrate that the highly facile methionine-oxaziridine modification approach can be simply applied to the synthesis of stable and site-specifically modified radiolabeled antibody–chelator conjugates with favorable pharmacokinetics for PET imaging.

## Introduction

Monoclonal antibodies (mAbs) in immunotherapy have revolutionized cancer treatment and prognosis in the past decade. Clinical use of antibodies could be further personalized by integrating molecular imaging to provide whole-body information on antibody biodistribution and antigen target expression (including heterogeneity) during therapy planning and treatment.^1^ Immuno-positron emission tomography (immunoPET) exquisitely combines the extraordinary targeting specificity of mAbs and the superior sensitivity of positron emission tomography (PET). ImmunoPET imaging has greatly increased our understanding of tumor heterogeneity and, ultimately, has played a vital role in guiding therapy treatment. Radiometals are frequently used for ImmunoPET and are most commonly attached to antibodies using chelators.^1^

Traditionally, antibody–chelator immunoconjugates are created via stochastic conjugation between the primary amine side chain of lysine (Lys) residues and reactive esters or isothiocyanate functional groups, yielding amide or thiourea conjugates, respectively. However, while these methods are simple and easy to implement, the high abundance of lysine residues results in a lack of selectivity in number and location of conjugation, which risks modifying the antigen-binding regions of a mAb, hampering its immunoreactivity.^2,3^ The resulting heterogeneous mixtures of immunoconjugates often demonstrate suboptimal pharmacokinetics and decreased affinities for target antigens.^4−6^ Methods have been developed enabling production of well-defined site-specifically labeled radioimmumoconjugates: such approaches utilize (i) cysteine (Cys) engineering;^7,8^ (ii) the heavy chain glycan region of a mAb, incorporating azide-bearing sugars via chemoenzymatic methods to facilitate “click” conjugation;^4,9−13^ (iii) enzymatic methods employing sortase A,^14−16^ microbial transglutaminase;^17−19^ and (iv) incorporation of an azide-containing non-natural amino acid (AA) facilitating site-selective conjugation.^20^ Recently, we have demonstrated an elegant approach utilizing dibromomaleimide motifs to simultaneously attach deferoxamine (DFO) and sarcophagine (sar) at IgG hinge regions while rebridging the two cysteines.^21^ While each of these strategies has their clear advantages, enzyme-based methods, for example, require expensive enzymes, glycan-based methods can alter the Fc-binding properties of the mAb and they are restricted to intact antibodies,^12,22^ and Cys engineering methods often require multistep reactions to reduce and reoxidize/rebridge disulfide bonds, potentially leading to disulfide scrambling.^23^

Modification of naturally occurring AAs to achieve a high level of selectivity is intrinsically challenging. Such difficulties result from a lack of site-specificity, that is, a mixture of heterogeneous products resulting from modification of repeated functionalities such as in the case of Lys and Cys (from reduced disulfides) residues. Targeting low abundance amino acids provides higher chances of forming selective unique chemical handles. Moreover, in cases where modification of an intrinsic AA is not available, genetic incorporation of canonical AAs available for conjugation is a well-established technique.

Methionine (Met) occurrences are rare in proteins, and they are often found in buried hydrophobic pockets, making Met an excellent target for site-specific modification. Owing to the less reactive side chain of Met in comparison to Lys and Cys, only a handful of strategies have been reported for Met-selective modification.^24−28^ Most notably, the redox-based (ReACT) strategy utilizes oxaziridine reagents that convert the Met thioether side chain to a sulfimide conjugate in a single step.^24^ The reaction is rapid and can be performed at neutral pH and room temperature. While the ReACT method is highly selective, it was previously shown that stability of the conjugate varies with the location of modification in an antibody scaffold, with the hydrolytic stability inversely proportional to the solvent accessibility of the site.^29^ In a parallel study by Christian et al.,^30^ it was demonstrated that stability of the conjugate can be vastly increased by incorporation of electron-donating *N*-substituents on the oxaziridine. Lin et al. have recently reported the [^18^F]F-labeling of Met residues in peptides and bovine serum albumin using the copper-mediated alkyne–azide coupling (CuAAC) reaction with alkyne-bearing modified Met sulfimide conjugates.^31^

A major disadvantage of IgG1 antibodies (∼150 kDa) as imaging probes is their long circulation time, leading to the requirement for significant time (1 day to 1 week) between administration/injection of the tracer and imaging protocols, to optimize target–to–background contrast in resultant images. In contrast, while monovalent antigen binding fragments (Fab) (∼50 kDa) typically have lower affinities compared to their full length IgG counterparts,^32^ visualization of targeted tissues is facilitated by rapid blood clearance, resulting in improved tumor-to-nontargeted-tissues contrast.^33^ Gallium-68 (*t*_1/2_ = 68 min; *E*_β+_ = 830 keV, 89%) is one of the leading β^+^-emitting radiometals for PET imaging due to its widespread availability from benchtop ^68^Ge/^68^Ga generators. The short half-life of ^68^Ga is well suited to the fast blood clearance of Fab fragments.^34,35^ We required a chelator that is capable of near quantitative radiochemical yields with [^68^Ga]Ga^3+^ at a near neutral pH, short incubation times, and mild temperature. We have previously shown that the siderophore, deferoxamine (DFO), is superbly suited for these purposes,^36^ and others have shown sufficient stability of the resulting ^68^Ga-DFO complex over short timeframes in a biological milieu.^37,38^

Intrigued by the highly selective and facile nature of the ReACT method, we explored this strategy for radiometal chelator–antibody conjugation. Herein, we describe the development of DFO-trastuzumab Fab conjugation using the ReACT platform and subsequent radiolabeling with [^68^Ga]Ga and assess the resulting [^68^Ga]Ga-DFO-trastuzumab Fab immunoconjugates in vitro and in vivo.

## Results

### Preparation of Trastuzumab DFO-Fab Conjugates

**Figure 1:**
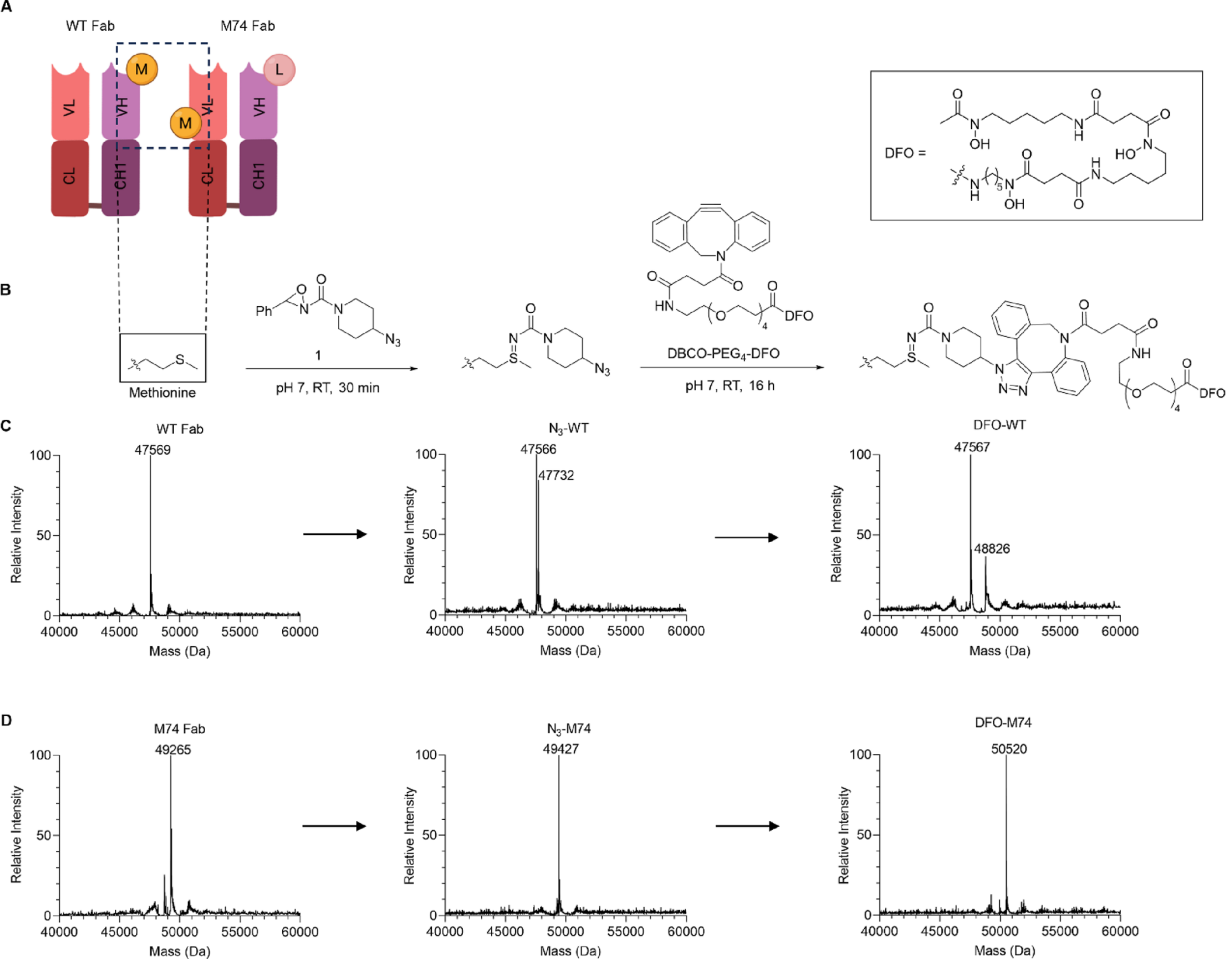
(A) Wild-type trastuzumab Fab fragment (WT Fab) and an engineered trastuzumab Fab (M74 Fab) containing T74 M and M107L mutations. (B) Preparation of DFO-Fab immunoconjugates via Met-oxaziridine conjugation followed by SPAAC with DFO-PEG_4_-DBCO. (C) Deconvoluted ESI-mass spectra of the WT-DFO conjugation reaction. Partial conjugation was observed, resulting in a mixture of unmodified WT-Fab and singly modified DFO-WT. (D) Deconvoluted ESI-mass spectra of the M74-DFO conjugation reaction. Full conversion of the unmodified Fab to singly modified DFO-M74 was observed. Full ESI-MS spectra are included in Figures S2–S7.

Our initial studies focused on modifying the wild-type (WT) trastuzumab Fab derivative. Oxaziridine-N_3_ (**1**) and DBCO-PEG_4_-DFO were prepared as previously described.^29,39^ WT Fab was generated by treating the intact IgG antibody with immobilized papain for 18 h. The Fab fragment was purified via an anion exchange column. The purity and structural integrity of Fab were assessed by SDS-PAGE (Figure S1) and intact MS. WT Fab contains a single Met (M107) (Figure 1A) in the heavy chain that could be modified upon treating with 20 equiv of **1**.^29^ Interestingly, in a separate contrasting study, Cotton et al. reported that 20% of the WT Fab N-terminus can be modified with just 5 equiv of oxaziridine.^40^ Incubation of the WT Fab (50 μM) with 20 equiv of **1** for 30 min at rt resulted in WT Fab functionalization to yield N_3_-WT Fab. Peptide mapping using a trypsin digest confirmed modification of M107 in the heavy chain (Figure S8). However, under these conditions, only partial conjugation was observed by ESI-MS (Figure 1C). Increasing the equivalence of **1** (30 and 50 equiv) used or increasing the reaction time to 1 h improved consumption of the starting Fab; however, a second species with *m*/*z* corresponding to dual modification of the Fab was observed (Figures S9–S11). We hypothesize that this may be due to undesired labeling of the N-terminus. To minimize dual conjugation, we employed 20 equiv of **1** for 30 min for DFO functionalization. The singly modified N_3_-WT Fab was further functionalized with DBCO-PEG_4_-DFO **2** (10 equiv) via the strain-promoted azide–alkyne cycloaddition (SPAAC) to yield a mixture containing both unmodified WT Fab and DFO-WT Fab (Figure 1C). No additional purification was performed, and DFO-WT-Fab was assessed as a mixture in further experiments. The suboptimal conjugation results were likely related to the limited solvent accessibility of M107. DFO-WT-Fab was recovered in 77% yield.

In light of the unsatisfactory conjugation results with the WT Fab, we turned our attention to modifying an engineered derivative containing a solvent-accessible Met available for conjugation. The Fab scaffold has previously been thoroughly scanned for suitable Met mutations that allow for high conjugation efficiency and stable conjugates.^29^ Guided by this prior work, we utilized the lead candidate, which contained the mutations heavy chain (HC).M107L and light chain (LC).T74 M (Figure 1A). The engineered Fab (M74 Fab) was successfully expressed in **Escherichia coli** and purified first using a protein A affinity column followed by size exclusion column chromatography. Purified M74 Fab was characterized by SDS-PAGE (Figure S1) and intact MS (Figure S2).

Only 15 equiv of **1** was required to modify M74 Fab, and full conversion to N_3_-M74 Fab was observed by ESI-MS after 30 min (Figure 1D). In contrast, no evidence of modification was observed under these conditions with the WT Fab. Further reaction of N_3_-M74 Fab with DBCO-PEG_4_-DFO yielded the DFO-M74 Fab conjugate as a single well-defined species as indicated by SDS-PAGE (Figure S1) and ESI-MS (Figure 1D). After purification, DFO-M74 was recovered in 79% yield.

### Radiolabeling and Serum Stability

**Figure 2:**
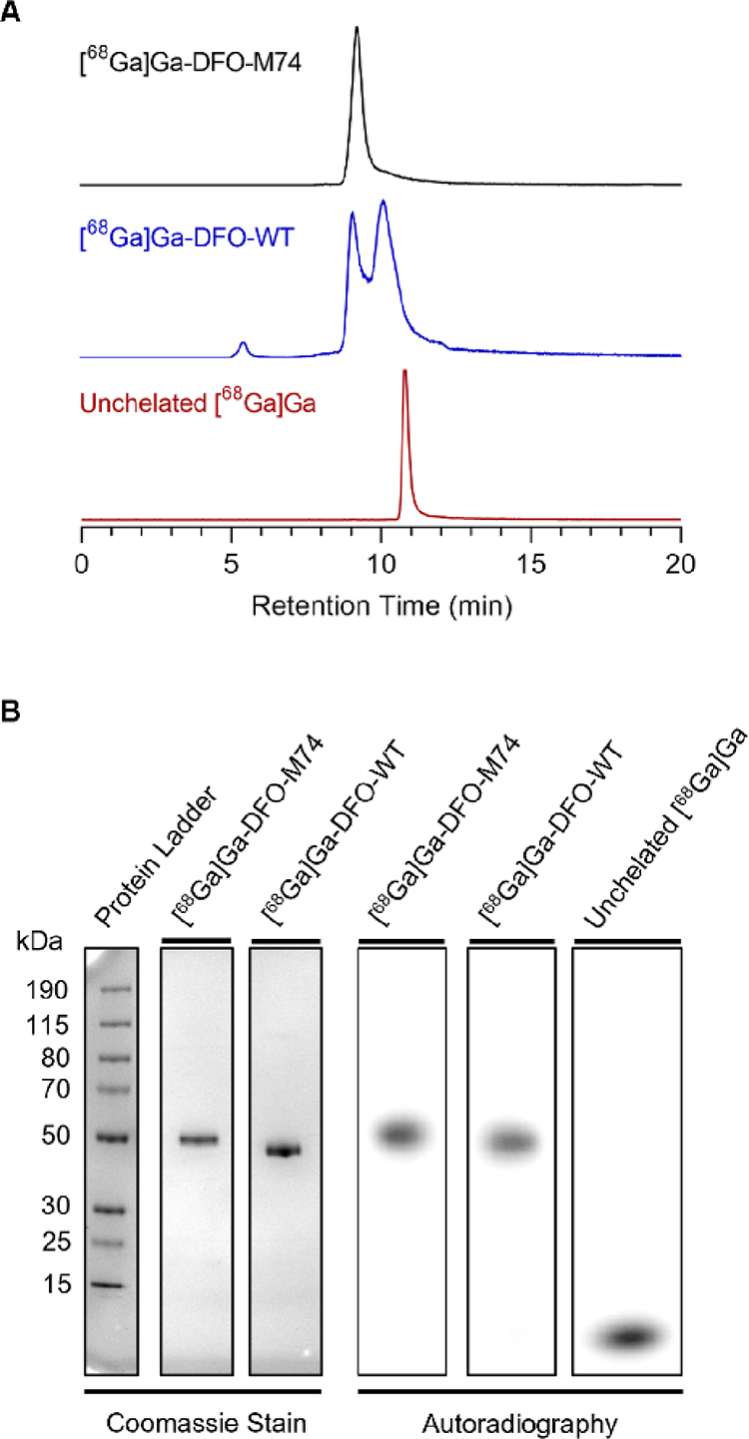
Characterization data for the synthesis of [^68^Ga]Ga-DFO-M74 and [^68^Ga]Ga-DFO-WT. (A) SE-HPLC radiochromatograms. (B) SDS-PAGE bright view image (left) and autoradiography (right); full SDS-PAGE images are included in Figure S12.

Both DFO-WT and DFO-M74 were radiolabeled with [^68^Ga]Ga^3+^. The conjugates (∼150 μg in 60 μL of aqueous 0.2 M NH_4_OAc solution) were combined with a solution containing [^68^Ga]Ga^3+^ (30 MBq, in 75 μL of aqueous 0.1 M HCl) and an aqueous solution of NH_4_OAc (2 M, 40 μL), followed by incubation at 37 °C for 15 min and subsequent analysis by size exclusion (SE)-HPLC (Figure 2A). The [^68^Ga]Ga-DFO-M74 conjugate eluted at 9.08 min while unreacted/unchelated [^68^Ga]Ga^3+^ eluted at 10.82 min. This crude product was simply purified using a Zeba spin desalting column (7K MWCO, 0.5 mL, 2 × 2 min), to yield [^68^Ga]Ga-DFO-M74 in a 36.4 ± 3.8% radiochemical yield (*n* = 5) (non-decay corrected) and in >99% radiochemical purity as observed by SE-HPLC. In contrast, the SE-HPLC analysis of Zeba spin-purified [^68^Ga]Ga-DFO-WT afforded multiple signals at 5.42, 9.08, and 10.10 min. The earlier retention time of the species eluting at 5.42 min indicates a larger molecular-weight species and is likely due to aggregation of [^68^Ga]Ga-DFO-WT. We further postulate that the two closely eluting signals at 9.08 and 10.10 min also correspond to [^68^Ga]Ga-DFO-WT, potentially in its native form and also at least partially denatured. We suggest that this aggregation and denaturation phenomena observed for [^68^Ga]Ga-DFO-WT are a result of modification/derivatization of the WT Fab (vide infra). [^68^Ga]Ga-DFO-WT was recovered in 33.8 ± 2.8% radiochemical yield (*n* = 5) (non-decay corrected).

To further probe these radiochemical reactions, [^68^Ga]Ga-DFO-M74 and [^68^Ga]Ga-DFO-WT were analyzed by nonreducing SDS-PAGE on bright-view imaging and autoradiography (Figure 2B). Similar to SE-HPLC analysis, both labeled conjugates alongside unchelated [^68^Ga]Ga^3+^ were observed in the crude reaction mixtures (Figure S12) for both [^68^Ga]Ga-DFO-M74 and [^68^Ga]Ga-DFO-WT. Following Zeba spin treatment, purified radiolabeled conjugates only showed ^68^Ga radioactive signal coinciding with stained protein band corresponding to a molecular weight of ∼50 kDa (Figure 2B). In contrast to the SE-HPLC analysis, only a single signal was observed for [^68^Ga]Ga-DFO-WT under the denaturing conditions of SDS-PAGE. This is consistent with our hypothesis that the multiple signals observed in the radio-SE-HPLC chromatogram for [^68^Ga]Ga-DFO-WT arise from aggregation and protein denaturation as a direct result of derivatization/modification.

Serum stability studies were carried out on [^68^Ga]Ga-DFO-M74 and [^68^Ga]Ga-DFO-WT to assess their stability in biological milieu prior to in vitro and in vivo studies: the radiolabeled immunoconjugates were incubated in human serum at 37 °C and aliquots were analyzed by SE-HPLC over 4 h (Figure S13). SE-HPLC radiochromatography analysis of [^68^Ga]Ga-DFO-M74 indicated that 95% of the conjugate remained intact after 4 h while 93% of [^68^Ga]Ga-DFO-WT remained intact. This size exclusion chromatography method did not enable distinction of radiometabolite products; it is possible that they arise as a result of instability of the ^68^Ga-labeled complex, instability of the sulfimide linkage, or even degradation of protein components by serum proteases.

### In Vitro Characterization of [^68^Ga]Ga-DFO-M74 and [^68^Ga]Ga-DFO-WT

**Figure 3:**
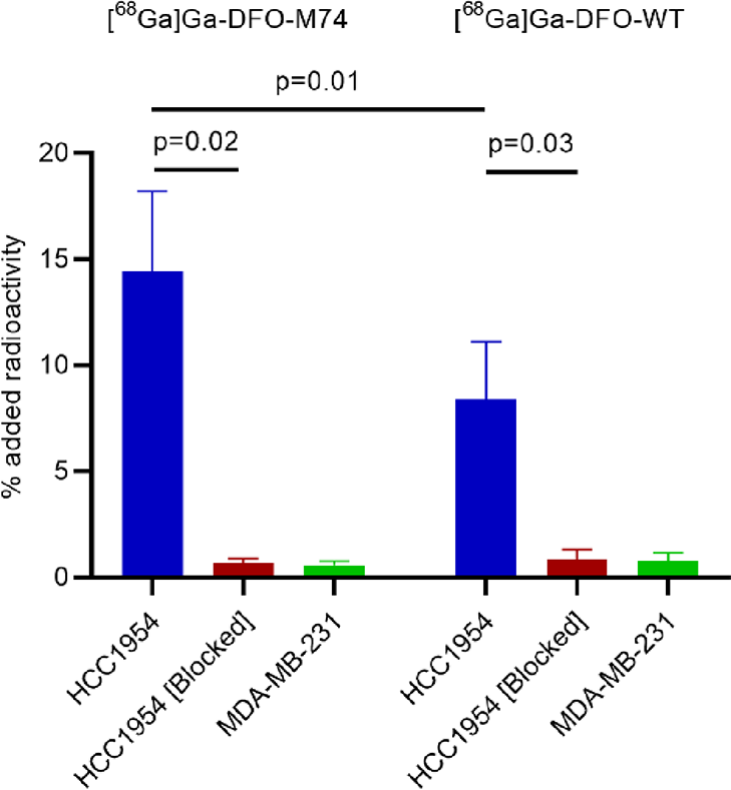
Uptake of [^68^Ga]Ga-DFO-M74 and [^68^Ga]Ga-DFO-WT in HER2-positive HCC1954 cells, HCC1954 cells in the presence of a 200-fold excess of trastuzumab, and HER2-deficient MDA-MB-231 cells. Studies were carried out in experimental triplicates (Mean ± SD, *n* = 3). See also Table S1.

To assess the relative binding of [^68^Ga]Ga-DFO-M74 and [^68^Ga]Ga-DFO-WT toward their HER2 target antigen, in vitro cell uptake experiments were carried out in HER2-positive HCC1954 cells and HER2-deficient MDA-MB-231 cells. Both [^68^Ga]Ga-DFO-M74 and [^68^Ga]Ga-DFO-WT showed significant uptake in HCC1954 cells with uptake inhibited by addition of an excess (200 equiv) of unmodified trastuzumab (Figure 3). Minimal uptake was observed in MDA-MB-231 HER2-deficient cells. Significantly, a 1.7-fold higher uptake was observed for [^68^Ga]Ga-DFO-M74 compared to [^68^Ga]Ga-DFO-WT in HCC1954 cells (*p* = 0.01). The observed difference is possibly related to the site of conjugation at M74 in [^68^Ga]Ga-DFO-M74 vs M107 in [^68^Ga]Ga-DFO-WT. The HC.M107 residue is located at the antigen binding region of the CDR H3 domain: modification and labeling at this site are likely to decrease the affinity of the antibody.^29^ It is, however, interesting to note that the binding affinity of [^68^Ga]Ga-DFO-WT toward HER2 is not completely ablated despite conjugation at its antigen-binding region. It is also possible that the likely lower stability of [^68^Ga]Ga-DFO-WT (which results in the formation of aggregates or denatured species, both presumably biologically inactive) leads to lower accumulation of [^68^Ga]Ga-DFO-WT in HER2-expressing cells. It should also be noted that in the case of [^68^Ga]Ga-DFO-WT, it is likely that unmodified WT Fab competed with radiolabeled and unlabeled DFO-WT-Fab for binding to HER2 receptors. This is yet another drawback of a bioconjugation approach that involves the formation of heterogeneous mixtures of products: it is experimentally very difficult to compare uptake and affinity of any single, defined species.

### In Vivo Evaluation of [^68^Ga]Ga-DFO-M74 and [^68^Ga]Ga-DFO-WT

**Figure 4:**
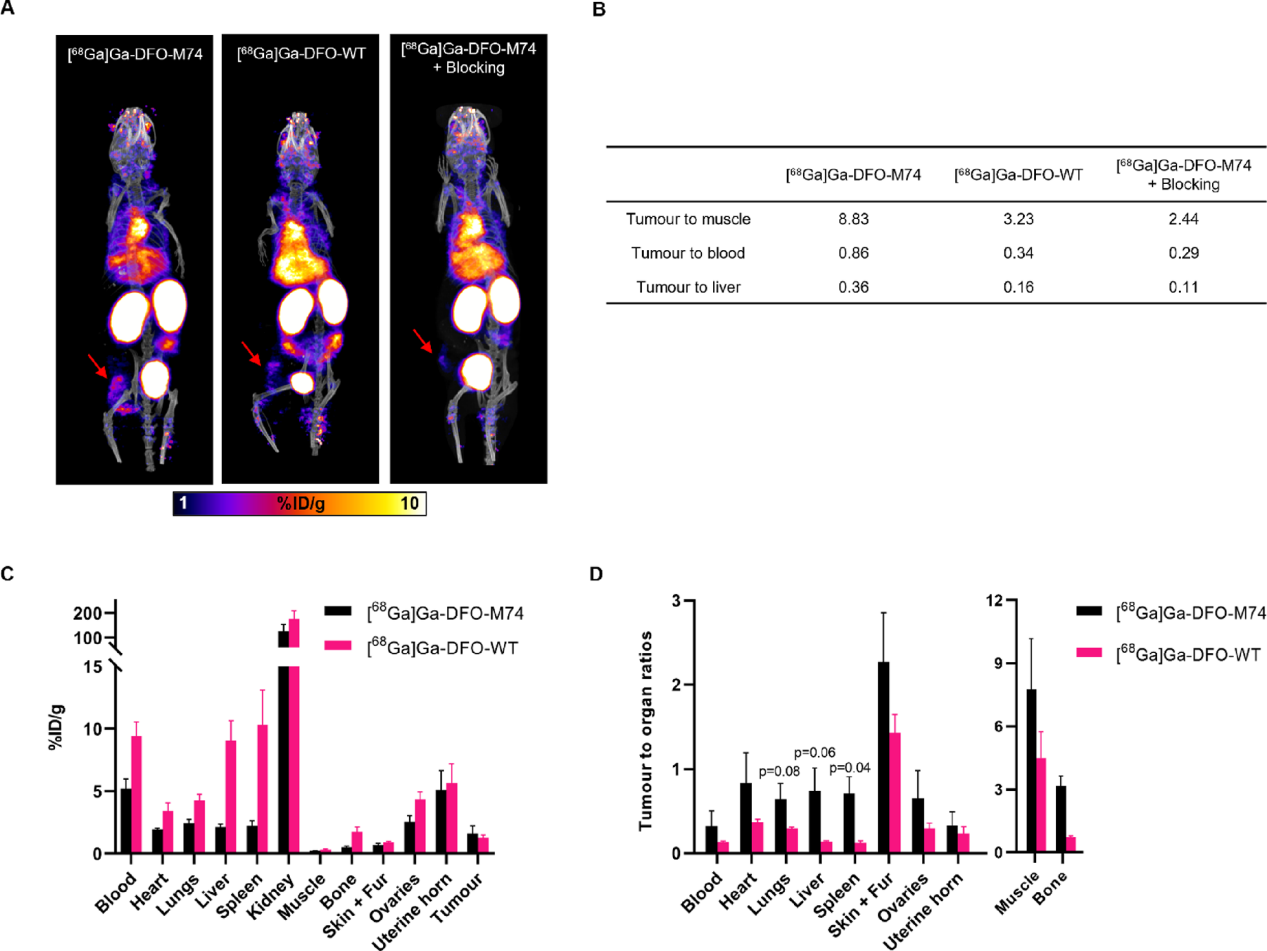
(a) PET/CT maximum intensity projections of HCC1954 tumor-bearing female NSG mice administered with [^68^Ga]Ga-DFO-M74 or [^68^Ga]Ga-DFO-WT or administered a blocking dose of trastuzumab prior to injection of [^68^Ga]Ga-DFO-M74, at 2 h p.i. of radiotracer, red arrows indicate HCC1954 tumors. (b) Tumor to organ/background ratios obtained from PET images (*n* = 1) (c) Ex vivo biodistribution of [^68^Ga]Ga-DFO-M74 and [^68^Ga]Ga-DFO-WT in HCC1954 tumor-bearing mice at 2 h p.i. (*n* = 3), see also Table S2. (d) Selected ex vivo biodistribution tumor to organ ratio of [^68^Ga]Ga-DFO-M74 and [^68^Ga]Ga-DFO-WT (Mean ± SD, *n* = 3), see also Table S3.

The biodistribution of [^68^Ga]Ga-DFO-M74 and [^68^Ga]Ga-DFO-WT were studied in NOD scid gamma (NSG) female mice bearing orthotopic HER2-positive HCC1954 tumors, using PET/CT imaging and ex vivo tissue biodistribution studies. [^68^Ga]Ga-DFO-M74 and [^68^Ga]Ga-DFO-WT (2.1–3.6 MBq) were each administered intravenously (via tail vein) to mice (*n* = 1 per bioconjugate), and PET/CT images were obtained over 4 h (Figures 4A and S14). PET/CT imaging showed that both [^68^Ga]Ga-DFO-M74 and [^68^Ga]Ga-DFO-WT accumulated rapidly at tumors (1.23 and 1.00%ID/g, respectively, at 2 h p.i.), which could be clearly delineated. However, clearance of [^68^Ga]Ga-DFO-WT from blood circulation and non-target organs was lower compared with that of [^68^Ga]Ga-DFO-M74 (Figure 4B). For example, at 2 h p.i., the tumor/muscle ratio was measured to be 3.23 for [^68^Ga]Ga-DFO-WT and 8.83 for [^68^Ga]Ga-DFO-M74. Interestingly, the amount of radioactivity in the liver was also higher for [^68^Ga]Ga-DFO-WT (6.18%ID/g) compared with [^68^Ga]Ga-DFO-M74 (3.37%ID/g), leading to tumor/liver ratios of 0.16 and 0.36, respectively. The increased tumor/muscle, tumor/blood, and tumor/liver ratios led to improved tumor contrast for [^68^Ga]Ga-DFO-M74 compared to [^68^Ga]Ga-DFO-WT. Lastly, to assess specificity of [^68^Ga]Ga-DFO-M74 using PET/CT imaging, one mouse was administered with a dose of trastuzumab 2 days prior to administration of [^68^Ga]Ga-DFO-M74. As predicted, compared to the animal injected with [^68^Ga]Ga-DFO-M74, significantly reduced tumor uptake was observed (0.56%ID/g) as well as a lower tumor/muscle ratio (2.44).

The ex vivo biodistribution of [^68^Ga]Ga-DFO-M74 and [^68^Ga]Ga-DFO-WT was also performed (*n* = 3 per group) at 2 h p.i. of tracers, mice were culled, and organs were dissected, weighed, and counted for radioactivity. Consistent with PET/CT imaging data, [^68^Ga]Ga-DFO-M74 and [^68^Ga]Ga-DFO-WT exhibited tumor uptakes of 1.6 ± 0.6 and 1.3 ± 0.2%ID/g, respectively (Figure 4C). Excretion of the tracers was largely renal, with the concentration in the kidneys measuring 125.6 ± 27.9%ID/g for [^68^Ga]Ga-DFO-M74 and 176.2 ± 33.4%ID/g for [^68^Ga]Ga-DFO-WT at 2 h p.i. Furthermore, the concentration of ^68^Ga radioactivity in the blood was higher for [^68^Ga]Ga-DFO-WT (9.4 ± 1.1%ID/g) compared with [^68^Ga]Ga-DFO-M74 (5.2 ± 0.8%ID/g, mean difference = 4.2 ± 0.8%ID/g, *p* = 0.008). Similarly, the concentration of ^68^Ga radioactivity in the liver was 9.0 ± 1.6%ID/g for [^68^Ga]Ga-DFO-WT, compared with 2.1 ± 0.2%ID/g for [^68^Ga]Ga-DFO-M74 (mean difference = 6.9 ± 0.9%ID/g, *p* = 0.016). This resulted in higher tumor-to-normal tissue ratios at 2 h for [^68^Ga]Ga-DFO-M74 compared to those for [^68^Ga]Ga-DFO-WT, consistent with PET/CT imaging data (Figure 4D). Importantly, skeletal uptake of the tracers were minimal: prior studies have shown that dissociated [^67^Ga]Ga^3+^ results in accumulation of activity in bones.^41,42^ In our study, the [Ga(DFO)] complex is sufficiently stable over the time course of the in vivo experiments. It is also noteworthy that the ovaries and uterine horn, which are healthy tissues known to express HER2,^43,44^ showed uptake of both tracers.

## Discussion

Conventional methods for the synthesis of antibody–chelator conjugates rely on modification of solvent accessible lysine residues, which often leads to ill-defined, heterogeneous mixtures that can exhibit suboptimal pharmacokinetics and decreased affinity for target receptors.^4−6^ The oxaziridine-based Met conjugation platform (ReACT) offers a rapid, simple method for site-specific antibody functionalization, presenting a clear advantage to cysteine-based labeling methods, which often requires reduction and reoxidation prior to conjugation.^5^ Utility of the ReACT platform for site-specific radionuclide incorporation was first demonstrated by Lin et al., combining oxaziridine chemistry with CuAAC, for the radiolabeling of peptides and bovine serum albumin with ^18^F.^31^ We improved this approach by employing the more facile copper-free SPAAC approach for the incorporation of DFO into trastuzumab Fab derivatives, avoiding the use of cytotoxic copper catalysts. In addition, we have combined this approach with the more cost-effective, generator-produced ^68^Ga, circumventing the need for expensive cyclotron-based infrastructure. These are the first examples of radiolabeled antibody-based conjugates that have been prepared using the ReACT platform.

We have demonstrated that the combination of antibody engineering to introduce a Met residue into a Fab fragment, followed by Met-functionalization using oxaziridine conjugate chemistry, enables homogeneous, site-specific radiolabeling using radiometals. We have used the siderophore chelator, DFO, for coordination of [^68^Ga]Ga^3+^, but envisage that other pairs of radiometallic ions/chelators could similarly be incorporated into Fab derivatives. Similarly, we also envisage that other Fab derivatives for other cancer and disease cell surface receptor markers could also be employed.

In vitro studies demonstrated that, while both new radioimmunoconjugates retained binding toward HER2-expressing HCC1954 cells, [^68^Ga]Ga-DFO-M74 exhibited higher affinity compared to [^68^Ga]Ga-DFO-WT. In vivo, the higher affinity and/or stability of [^68^Ga]Ga-DFO-M74 compared to [^68^Ga]Ga-DFO-WT led to faster blood clearance, lower accumulation in liver, and slightly higher tumor uptake. In combination, these factors resulted in improved contrast for [^68^Ga]Ga-DFO-M74; thus, we believe that there are clear advantages of using the M74 oxaziridine-modified Fab compared with the analogous WT Fab for PET imaging of HER2 expression.

The trastuzumab-derived Fab fragment has generated considerable interest for nuclear imaging due to its favorable pharmacokinetics profile. Its derivatives radiolabeled with ^68^Ga^34^ and longer-lived radioisotopes ^64^Cu (*t*_1/2_ = 12 h)^45,46^ or ^111^In (*t*_1/2_ = 2.8 d)^32,45,47^ have shown promise to be clinically useful for imaging HER2 expression. However, none of these derivatives are prepared site-specifically, and this may affect their binding affinities. Indeed, Rathore et al. reported a 10-fold decrease in binding affinity for the stochastically modified trastuzumab-Fab-NOTA (for ^68^Ga radiolabeling) compared to unmodified trastuzumab Fab.^34^ The authors reasoned that this could be due to the attachment of the chelator to K64 in the receptor binding site, which may mask receptor interactions. Such complications can be avoided via site-specific chelator conjugation through a judicious choice of modification site, and we have demonstrated that the ReACT platform can be easily applied for this application. While a high tumor-to-muscle ratio was achieved with [^68^Ga]Ga-DFO-M74, similar to values reported with ^111^In- and ^64^Cu-DOTA-Fab at 24 h p.i., the study was limited by the half-life of ^68^Ga and the relatively high amounts of radioactivity that remained in circulation at 2 h p.i.^45^ In this work, we have elected to use DFO to enable ^68^Ga-radiolabeling; however, other chelators such as NOTA or DOTA could enable applications with a wider range of radionuclides, including ^44^Sc, ^64^Cu, and ^111^In, which could complement the pharmacokinetics of the Fab fragments more fittingly.^48^ We are currently developing new oxaziridine-functionalized chelators for this purpose and comparing the bioconjugates of these with conventionally prepared bioconjugates containing mixtures of non-site-specifically labeled species.

## Conclusions

Antibodies modified site-specifically via the ReACT platform give rise to immunoconjugates that are well-defined and homogeneous. We took advantage of this approach in combination with the highly facile SPAAC to prepare two trastuzumab Fab conjugates modified with a DFO chelator. The resulting immunoconjugates DFO-WT and DFO-M74 were successfully radiolabeled with ^68^Ga to yield novel PET radiotracers [^68^Ga]Ga-DFO-M74 and [^68^Ga]Ga-DFO-WT. In vitro and In vivo studies revealed that [^68^Ga]Ga-DFO-M74 exhibited higher affinity toward HER2 receptors and demonstrated more favorable pharmacokinetics, resulting in improved contrast compared to [^68^Ga]Ga-DFO-WT. The promising results obtained with [^68^Ga]Ga-DFO-M74 opens up possibilities to explore the use of the ReACT platform for other radiometals/chelators and other Fab derivatives.

## Experimental Procedures

### Preparation of Trastuzumab Fab-DFO Immunoconjugates

Oxazridine-N_3_**1** and DBCO-PEG_4_-DFO were prepared as previously described.^29,39^ Trastuzumab Fab (5 mg) were incubated at 50 μM with **1** (15 equiv for M74, 20 equiv for WT) for 30 min at rt in PBS after which the reactions were quenched by addition of 400 mM l-Met (10 μL) and buffer exchanged into PBS using a 5 mL Zeba 7 kDa MWCO desalting column (1500*g*, 2 min). Then, 10 mol equiv of DBCO-PEG_4_-DFO (5 mM stock in DMF) was added, and the reaction mixtures were incubated at rt overnight. The conjugate was purified and buffer exchanged into ammonium acetate (0.1 M) using a PD-10 column and eluted in 0.5 mL fractions. Fractions containing protein were collected and further purified and buffer exchanged into ammonium acetate (0.1 M) by six cycles of spin filtration through Amicon Ultra-0.5 mL centrifugal filters (10 kDa MWCO, 17000*g*, 10 min). M74 Fab-DFO conjugate (4.89 mg) was recovered in 100 μL at a concentration of 967 μM while the WT Fab-DFO conjugate (4.3 mg) was recovered in 100 μL at a concentration of 881 μM. Samples for ESI-TOF MS analysis were prepared at 10 μM in 0.1 M ammonium acetate or in H_2_O (desalted using 0.5 mL Zeba 7 kDa MWCO desalting columns, 1500*g*, 2 min).

### Radiolabeling of Trastuzumab Fab-DFO Conjugates with [^68^Ga]Ga^3+^

[^68^Ga]Ga^3+^ was eluted from a GalliAd generator where ^68^Ge was attached to a tin dioxide column using 0.1 M HCl. ∼430 MBq [^68^Ga]Ga^3+^ is typically obtained in 1.1 mL and is used without further purification. Fab-DFO conjugates were diluted to 50 μM in NH_4_OAc (0.2 M). To 60 μL of this solution were added 40 μL of NH_4_OAc (2 M) and 75 μL of [^68^Ga]Ga^3+^ in HCl (0.1 M). The resulting solution was incubated at 37 °C for 15 min and purified using a Zeba spin 0.5 mL 7 kDa MWCO desalting column twice (1500*g*, 2 min). The reaction was then analyzed by SEC-HPLC, iLTC, and SDS-PAGE. iTLC-SG strips were spotted with 1 μL of radiolabeling mixture and developed in a mobile phase of citrate buffer (0.1 M, pH 5.5). For ^68^Ga-Fab-DFO, *R*_f_ < 0.1, and for unreacted ^68^Ga, *R*_f_ > 0.9. SEC-RadioHPLC: [^68^Ga]Ga-DFO-M74 had a retention time of 9 min while unchelated ^68^Ga eluted at 11.2 min; [^68^Ga]Ga-DFO-WT afforded species with retention times at 5.4 min (aggregated species), 9.1, and 10.1 min. Radiochemical purity determined by SEC-HPLC: [^68^Ga]Ga-DFO-M74: >99% at a specific activity of 84.2 MBq mg^–1^ or molar activity of 4.25 GBq μmol^–1^ and [^68^Ga]Ga-DFO-WT: >99% at a specific activity of 74.5 MBq mg^–1^ or molar activity of 3.63 GBq μmol^–1^.

#### SDS PAGE Analysis of Radioimmunoconjugates

Samples of radioimmunoconjugate (2 μL) and unchelated radiometal (2 μL) were diluted with water (10 μL) followed by addition of NuPAGE LDS sample buffer (4 μL), and the solutions were mixed thoroughly and loaded onto a 4–12% bis-tris protein gel along with a molecular weight marker. A constant voltage (180 V, 40 min) was applied to the gel after which it was imaged via autoradiography followed by bright light imaging (Coomassie blue staining).

### Serum Stability of [^68^Ga]Ga-DFO-M74 and [^68^Ga]Ga-DFO-WT

^68^Ga-labeled radioimmunoconjugates were prepared as above, and radiochemical yields and purities were assessed using iTLC and SEC-HPLC. A sample of radiolabeled compound was added to serum, in a ratio of one-part radiolabeled solution to four parts of serum by volume, and incubated at 37 °C. Aliquots were taken for SE-HPLC analysis at *t* = 1, 2, 3, and 4 h.

### Target Receptor Affinity of [^68^Ga]Ga-DFO-M74 and [^68^Ga]Ga-DFO-WT

[^68^Ga]Ga-DFO-M74 and [^68^Ga]Ga-DFO-WT were prepared as above and buffer exchanged into HBSS buffer using Zeba spin 0.5 mL 7 kDa MWCO desalting columns; the radioimmunoconjugates were then diluted with HBSS to a final concentration of 1.5 μM prior to uptake studies. Once harvested after trypsinization, HCC1954 and MDA-MB-231 cells were washed once with PBS and resuspended in HBSS with 0.2% BSA to give 1 × 10^6^ cells per tube in 500 μL. [^68^Ga]Ga-DFO-M74 and [^68^Ga]Ga-DFO-WT (11 μL, 1.5 μM, 0.8 μg in HBSS) were added to each tube. For blocking conditions, unmodified Trastuzumab (0.5 mg, 25 μL in saline) was added to the tubes and incubated at rt for 5 min prior to addition of the radioimmunoconjugates. Tubes were then incubated at 37 °C for 1 h. The cells were isolated by centrifugation at 400*g* for 5 min, aspirating the supernatant, and resuspended in PBS. The cells were washed twice by centrifuging at 400*g* for 5 min and aspirating the supernatant. The radioactivity associated with cell pellets and supernatant was then counted using a gamma counter.

### In Vivo Assessment of [^68^Ga]Ga-DFO-M74 and [^68^Ga]Ga-DFO-WT

All animal experiments were ethically reviewed by an Animal Welfare & Ethical Review Board at King’s College London and carried out in accordance with the Animals (Scientific Procedures) Act 1986 (ASPA) UK Home Office regulations governing animal experimentation.

#### Mammary Fat Pad Breast Cancer Tumor Establishment

Seven to nine-week-old female NOD scid gamma (NSG) (NOD.Cg-Prkdc^scid^ Il2rg^tm1WjI^/SzJ) mice (18–25 g) were obtained from Charles Rivers Laboratories. Animals were housed in ventilated cages, given food and water ad libitum, and allowed to acclimatize for approximately 1 week before tumor cell inoculation. Approximately 1.5 × 10^6^ HCC1954 cells in a 100 μL cell suspension of a 1:1 mixture of PBS/BD Matrigel (BD Biosciences) were injected subcutaneously in the mammary fat pad between the fourth and fifth nipples in the left flank. Experiments were performed approximately 3 weeks after the injection of cancer cells. Mice bearing mammary fat pad xenografts were randomly assigned to three groups (*n* = 3–4/group, tumor volume, ∼100–150 mm^3^). In the blocking group, 48 h before the scanning/biodistribution studies, trastuzumab (0.5 mg in 200 μL of PBS) was administered via the tail vein of anaesthetized mice.

#### PET/CT Imaging and Reconstruction

Preclinical PET/CT images were acquired using a NanoScan PET/CT scanner (Mediso, Budapest, Hungary) with mice under 0.8–1.5% isoflurane in oxygen anesthesia and warmed to 37 °C for the duration of the experiment. Mice were administered radiolabeled Fab fragments (∼50 μg, ∼ 2.1–3.6 MBq) in 200 μL of PBS via intravenous tail injection. Dynamic PET scans were acquired for up to 4 h post injection, followed by a CT scan for anatomical visualization (480 projections; helical acquisition; 55 kVp; 600 ms exposure time). PET/CT data sets were reconstructed using a Monte Carlo-based full-3D iterative algorithm (TeraTomo, Mediso) with 4 iterations, 6 subsets, and 0.4 mm isotropic voxel size. Images were coregistered and analyzed using VivoQuant v.3.0 (Invicro). Regions of interest (ROIs) were delineated for the PET activity quantification in specific organs. Uptake in each ROI was expressed as a percentage of injected dose per gram of tissue (% ID/g).

#### Biodistribution Studies

Mice (*n* = 3/group) were administered with similar amounts of conjugates that were used for imaging study and were maintained under anesthesia until the chosen time point (*t* = 2 h p.i., based on the imaging results). Mice were culled by cervical dislocation, and organs were dissected, weighed, and gamma-counted along with standards prepared from the corresponding sample of injected radiolabeled conjugates. Conjugate uptake was calculated as a percentage of injected dose per gram (% ID/g) of tissue.

## Supporting Information Available

Experimental descriptions, full SDS-PAGE data, ESI-MS, HPLC chromatograms, cell uptake study data, PET/CT image analysis, and ex vivo biodistribution data (PDF)
