# Supplementary material for: Site-Specific 68Ga Radiolabeling of Trastuzumab Fab via Methionine for ImmunoPET Imaging
Source: Bioconjug Chem. 2023 Sep 26;34(10):1802–10. doi: 10.1021/acs.bioconjchem.3c00344 (PMC10587866; doi:10.1021/acs.bioconjchem.3c00344)
Supplement: Supplementary file 1 — bc3c00344_si_001.pdf [file bc3c00344_si_001.pdf]

Site-Specific ^{68}Ga Radiolabelling of Trastuzumab Fab *via* Methionine for ImmunoPET Imaging

Thomas T. C. Yue,[1,2] Ying Ge,[1] Francesco A. Aprile,[3] Michelle T. Ma,[2] Truc T. Pham*,[2] and Nicholas J. Long*[3]

[1] Department of Chemistry, Molecular Sciences Research Hub, Imperial College London, White City Campus, Wood Lane, London W120BZ, UK.

Email: n.long@imperial.ac.uk

[2] *School of Biomedical Engineering and Imaging Sciences, King's College London, 4th Floor Lambeth Wing, St. Thomas' Hospital, London SE17EH, UK*

Email: truc.pham@kcl.ac.uk

[3] Department of Chemistry and Institute of Chemical Biology, Molecular Sciences Research Hub, Imperial College London, White City Campus, Wood Lane, London W120BZ, UK.

Supporting Information

Experimental Section

Materials and Instrumentation 2

Preparation of Trastuzumab M74 Fab fragment 2

Preparation of Trastuzumab WT Fab fragment 3

Peptide mapping analysis 3

Cell culture for HER2 *in vitro* binding studies 4

Supplementary Figures and Data 5

References 16

Materials and Instrumentation

Chemicals were purchased from Merck Chemicals Ltd or Fluorochem Ltd unless otherwise specified and used without further purification. Other solvents were purchased from VWR International and used without further purification. 18.2 MΩ water was used to prepare all buffers and aqueous solutions. Oxazridine-N_3 **1** and DBCO-PEG$_4$-DFO were prepared as previously described.[1,2] Trastuzumab was obtained as the biosimilar Herzuma in solution (21 mg/mL) from the Pharmacy Department at Guy's and St. Thomas' NHS Trust, London. Fresh human serum was obtained from a healthy volunteer. PD-10 size exclusion columns were purchased from GE Healthcare UK Ltd. Zeba™ Spin Desalting columns, 7 kDa MWCO, 0.5 mL, 5 mL and 10 mL were purchased from Life Technologies Limited, UK. Amicon Ultra 0.5 mL centrifugal filters (10 kDa MWCO), OverExpress™ C43 (DE3) Chemically Competent Cells and Overnight Express™ Instant TB Medium were purchased from Merck Chemicals Ltd. Instant thin layer chromatography (iTLC-SG) were obtained from Varian Medical Systems UK, Ltd. NuPAGE™ 4 to 12 %, Bis-Tris, 1.0 mm mini protein gels were obtained from Life Technologies Limited, UK. We thank S. Elledge (University of California, San Francisco) for providing the trastuzumab M74 Fab expression vector. Protein A affinity chromatography and size exclusion chromatography were performed on an AKTA pure 25L purification system using an HiTrap Protein A HP 5 mL column (Cytiva) and HiLoad 16/600 Superdex 75 pg column (GE Healthcare), respectively. Protein concentration was determined using a Thermo Scientific Nanodrop One spectrophotometer. High resolution mass spectrometry data and intact protein mass spectrometry were recorded by Mass Spectrometry Service, Imperial College London using a Waters LCT Premier (ES-TOF) spectrometer. HPLC-MS analysis were conducted on a Waters 515 HPLC pump, 2998 photodiode array detector and 3100 mass detector using either a Waters XSelect CSH C18 Column, 130 Å, 5 µm, 4.6 mm x 100 mm column or a Waters XBridge BEH C18 Column, 130 Å, 5 µm, 4.6 mm x 100 mm column. Gamma counting was performed using a Wallac 1282 Compugamma Universal Gamma Counter. Peptide mapping analysis was performed by the BSRC Mass Spectrometry and Proteomics facility at the University of St Andrews. Analytical size exclusion radioHPLC traces were acquired using an Agilent 1260 series HPLC system with an in-line radioactivity detector (LabLogic Systems Limited 1"NaI/PMT Detector). A BioSep™ SEC-s2000 column (5 µM, 145 Å, 300 x 7.8 mm) was used with a mobile phase of PBS supplemented with sodium ethylenediamine tetraacetate (2 mM) at a flow rate of 1 mL/min. iTLC strips were visualised using a Lab Logic Dual Scan-RAM radio-TLC/HPLC Scanner. SDS PAGE gels were visualised using an Invitrogen iBrightFL1000 imaging system (bright view) and GE Healthcare Amersham Typhoon imager (autoradiography).

Preparation of Trastuzumab M74 Fab fragment

Trastuzumab Fab plasmid vector containing HC.M107L, LC.T74M mutations was kindly provided by Elledge et al. (University of California, San Francisco). The protein sequences of light chain and heavy chain are shown below. Fab fragment was expressed in OverExpress™ C43 (DE3) chemically competent cells (Merck) on a 2 L scale. C43 (DE3) cells were grown in Overnight Express™ Instant TB Medium (Merck) at 37 ^oC with shaking at 190 rpm until the optical density at 600 nm reached 0.75 after which the temperature was lowered to 30 ^oC and further incubated for 16 h. The cells were harvested by centrifugation (5000 *g*, 20 min), resuspended in 20 mM Phosphate buffer, and lysed by sonication (500 W, 20 % amplitude, 15 s on 45 s off for 10 min). Cell lysate was collected by centrifugation (38758 *g*, 30 min) and the supernatant was pass through a 0.45 µm syringe filter prior to purification. Purification: 1) Cell lysate was first applied to a 5 mL HiTrap Protein A HP column (binding buffer: Phosphate-buffered saline (PBS), elution buffer: 0.1 M sodium citrate buffer pH 3) on an AKTA pure

25L purification system followed by 2) size exclusion chromatography using a HiLoad 16/600 Superdex 75 pg column (GE Healthcare, UK) (Elution buffer: PBS). Samples for ESI-TOF MS analysis were prepared at 10 μM in 0.1 M ammonium acetate or in H_2O (desalted using 0.5 mL Zeba 7-kDa desalting columns, 1500 *g*, 2 min). Samples were additionally analysed by SDS-PAGE.

Heavy Chain Sequence:

EISEVQLVESGGGLVQPGGSLRLSCAASGFNIKDTYIHWVRQAPGKGLEWVARIYPTNGYTRYADSV
KGRFTISADTSKNTAYLQMNSLRAEDTAVYYCSRWGGDGFYALDYWGQGTLVTVSSASTKGPSVFP
LAPSSKSTSGGTAALGCLVKDYFPEPVTVSWNSGALTSGVHTFPAVLQSSGLYSLSSVVTVPSSSLG
TQTYICNVNHKPSNTKVDKKVEPKSCDKTHTGGSGSHHHHHH

Light Chain Sequence:

SDIQMTQSPSSLSASVGDRVTITCRASQDVNTAVAWYQQKPGKAPKLLIYSASFLYSGVPSRFSGSR
SGTDFTLMISSLQPEDFATYYCQQHYTTPPTFGQGTKVEIKRTVAAPSVFIFPPSDEQLKSGTASVVC
LLNNFYPREAKVQWKVDNALQSGNSQESVTEQDSKDSTYSLSSTLTLSKADYEKHKVYACEVTHQG
LSSPVTKSFNRGEC

Preparation of Trastuzumab WT Fab fragment

Following a literature procedure.[3] Trastuzumab was supplied as the biosimilar Herzuma in solution (21 mg/mL) from the Pharmacy Department at Guy's and St. Thomas' NHS Trust, London. Briefly, Trastuzumab IgG (15 mg, final concentration: 67 μM) was reacted with 1.7 % wt/wt of immobilised papain (250 μg/mL) for 22 h at 37 ^{o}C in a buffer containing 20 mM sodium phosphate monobasic, 10 mM disodium EDTA and 80 mM cysteine HCl (pH 7), cysteine HCl was added immediately before digestion. After digestion, Tris.HCl buffer was added (pH 7.5, 2 mL) and the mixture centrifuged (3200 *g*, 10 min). The supernatant containing the Fab and Fc fragments were collected. Purification was conducted using a 20 mL HIPrep Q HP anion exchange column on an AKTA Pure 25L purification system (binding buffer: 50 mM Tris-HCl pH 8 buffer, elution buffer: 1 M NaCl in 50 mM Tris HCl pH 8 buffer). Samples for ESI-TOF MS analysis were prepared at 10 μM in 0.1 M ammonium acetate or in H_2O (desalted using 0.5 mL Zeba 7-kDa desalting columns, 1500 *g*, 2 min). Samples were additionally analysed by SDS-PAGE.

Peptide mapping analysis

Proteins were diluted to 10 μM with ammonium bicarbonate and digested with trypsin. The samples were analysed by nanoLCMSMS on a Sciex 5600 QTof mass spectrometer coupled with Sciex Eksigent 425 nanoLC. The LC was configured in trap elute format, with Waters acuity UPLC M class symmetry trap column 100 Å 5 μm 2 G 180 μm x 20 mm trap and Waters acquity UPLC Mclass HSS1 1.8 μm 75 μm x 150 mm column, both Waters. 5μL of sample was injected onto the trap in 15 μL/min of loading buffer (0.05 % Trifluoroacetic acid in water), and run for 5 min. The trap was switched in line with the analytical column and the sample eluted at a gradient over 35 min (A = 100 % water with 0.1 % formic acid, B = 20 % water 80 % acetonitrile, 0.1 % formic acid, 2 % A to 1 min, linear to 40% A over 25 min, linear to 95 % A over 4 min, hold for 1 min, linear back to 2 % A, and re-equilibrate for 4 min). The flow from the column was sprayed directly into the nanospray orifice at a voltage of 2300 V positive ionisation. Mass spectrometry data was collected from 350 to 1250 m/z for the survey scan with the top 20 most intense peaks selected by Data Dependant Acquisition conditions for MSMS with collision induced dissociation (CID) fragmentation. Raw data was exported and extracted using ms convert (ProteoWizard). The data searched using Mascot search engine (MatrixScience) against an internally

generated database of 6700 protein sequences. Settings were 20 ppm on the MS and 0.1Da on the MSMS data, with variable oxidation of methionine. For the identification of the Trastuzumab modification (C(6) H(9) N(5) O 167.1686 Da) was set as a variable modification on methionine residues.

Cell culture for HER2 *in vitro* binding studies

The human breast cancer cell lines HCC1954 (cultured in RPMI-1640) and MDA-MB-231 (cultured in DMEM low glucose (1 g/L)) were purchased from American Type Culture Collection. Growth media were supplemented with 10 % foetal bovine serum (FBS), 100 units/mL penicillin, 100 µg/mL streptomycin, and 2 mM L-glutamine. Cell lines were harvested twice weekly using a formulation of 0.25 % trypsin/0.53 mM EDTA in Phosphate Buffered Salt (PBS) Solution without calcium and magnesium, which was then neutralised with the appropriate medium containing FBS. Cells were maintained in a humidified chamber containing 5 % CO_2 at 37 ^{o}C.

Supplementary Figures and Data

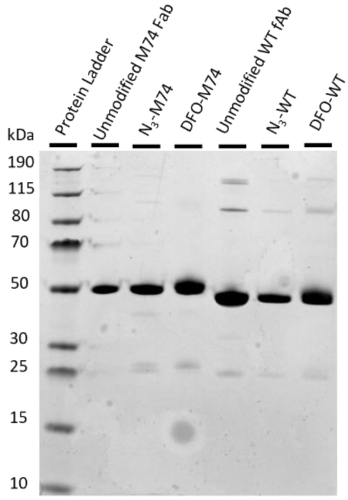

Figure S1: SDS-PAGE analysis of the conjugation reaction between Trastuzumab Fab (M74 and WT) with Ox-N_3 **1** (lane 3 and 6) and the subsequent reaction with DBCO-PEG_4-DFO (Lane 4 and 7)

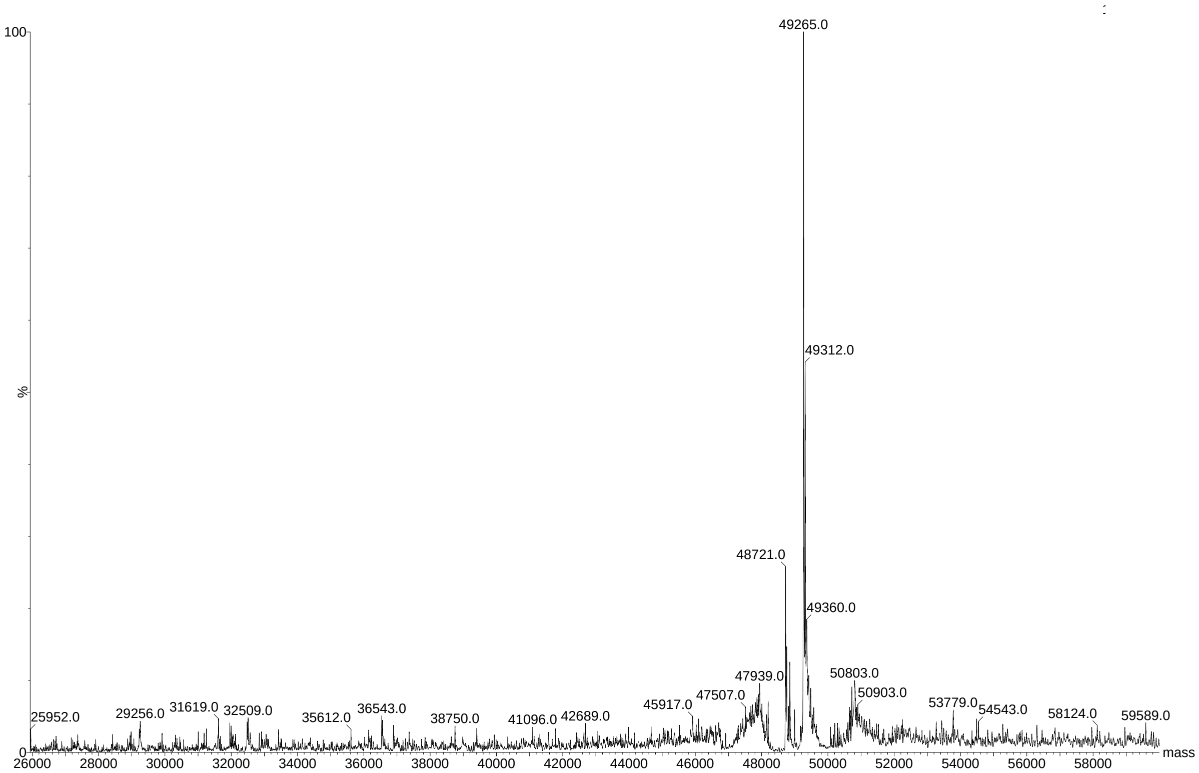

Figure S2 ESI deconvoluted mass spectrum of unmodified trastuzumab M74 Fab

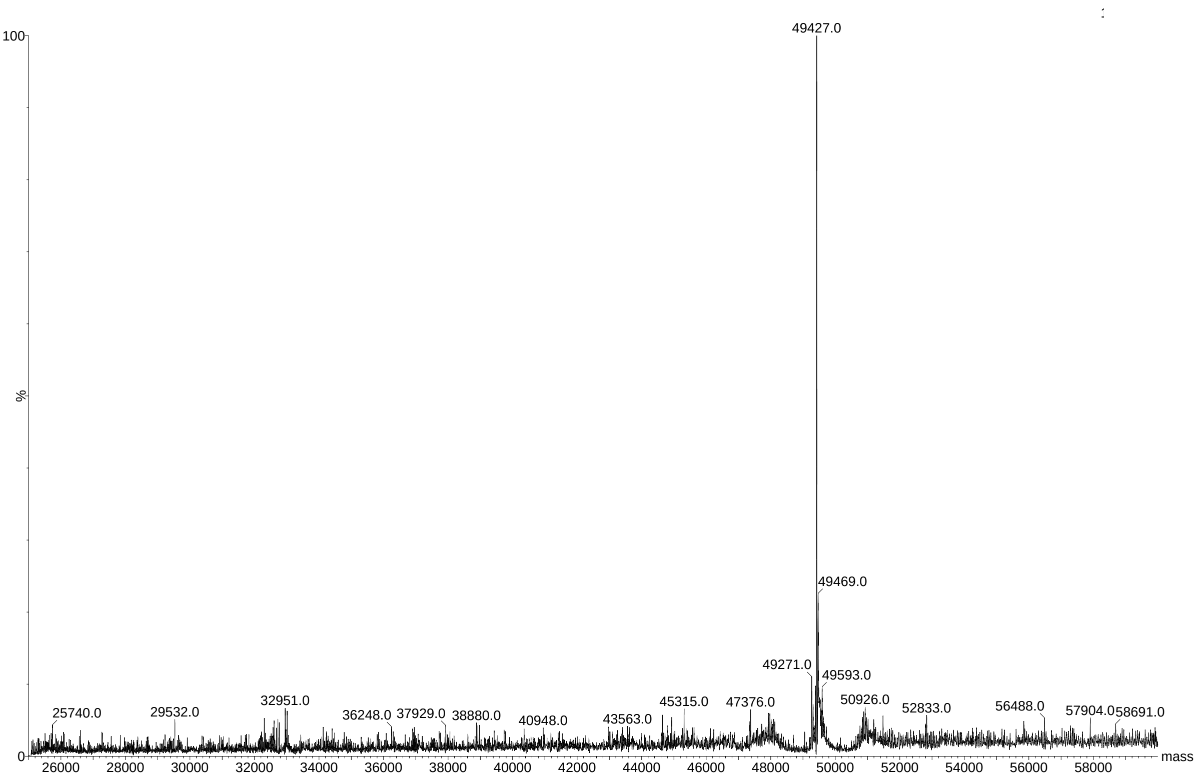

Figure S3 ESI-deconvoluted mass spectrum of the reaction between trastuzumab M74 Fab and 15eq. Ox-N_3 **1** to yield N_3-M74

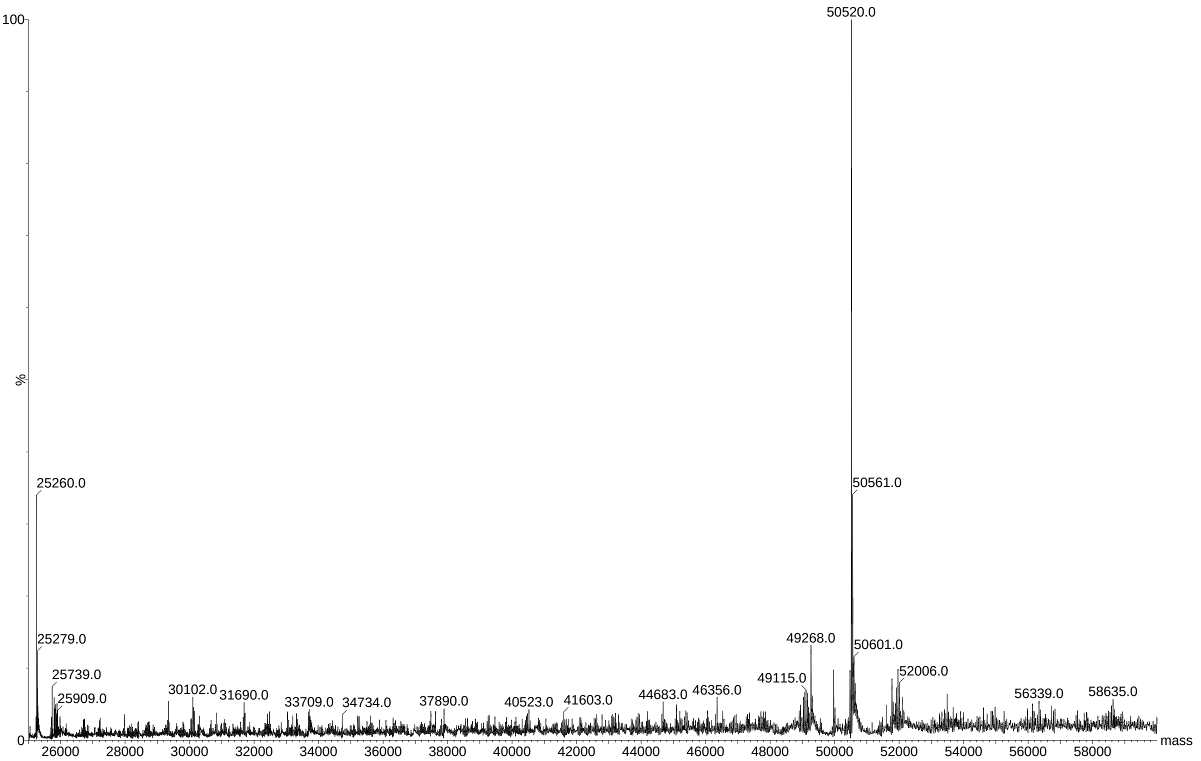

Figure S4 ESI-deconvoluted mass spectrum of the reaction between trastuzumab N_3-M74 and DBCO-PEG$_4$-DFO to yield DFO-M74

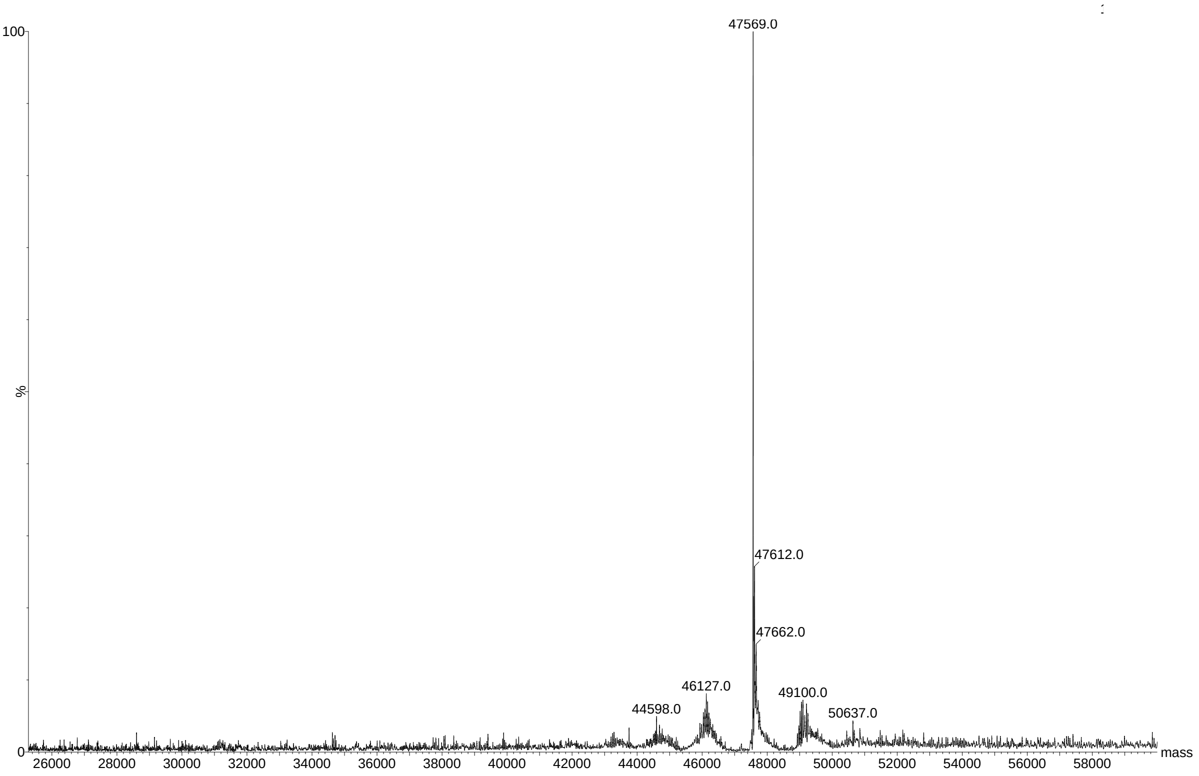

Figure S5 ESI-deconvoluted mass spectrum of unmodified trastuzumab WT Fab

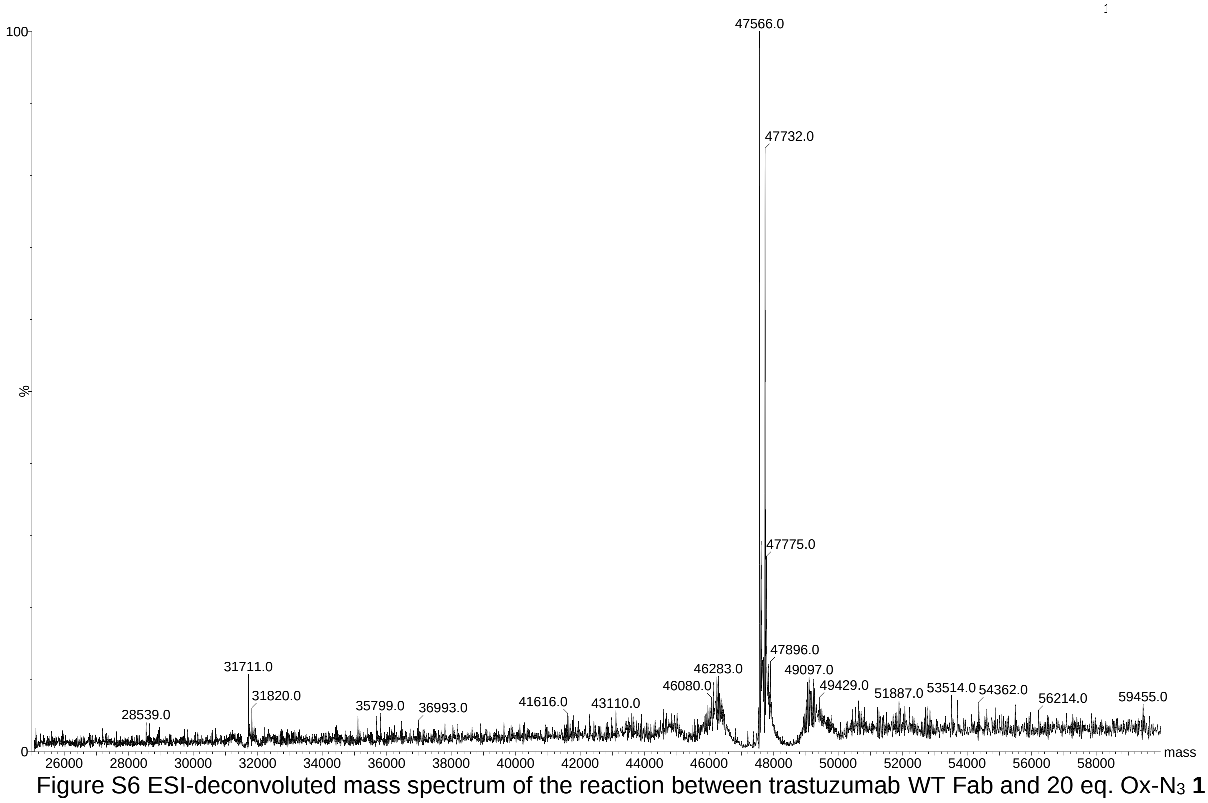

Figure S6 ESI-deconvoluted mass spectrum of the reaction between trastuzumab WT Fab and 20 eq. Ox-N_3 **1** to yield a mixture containing unmodified WT Fab and N_3-WT.

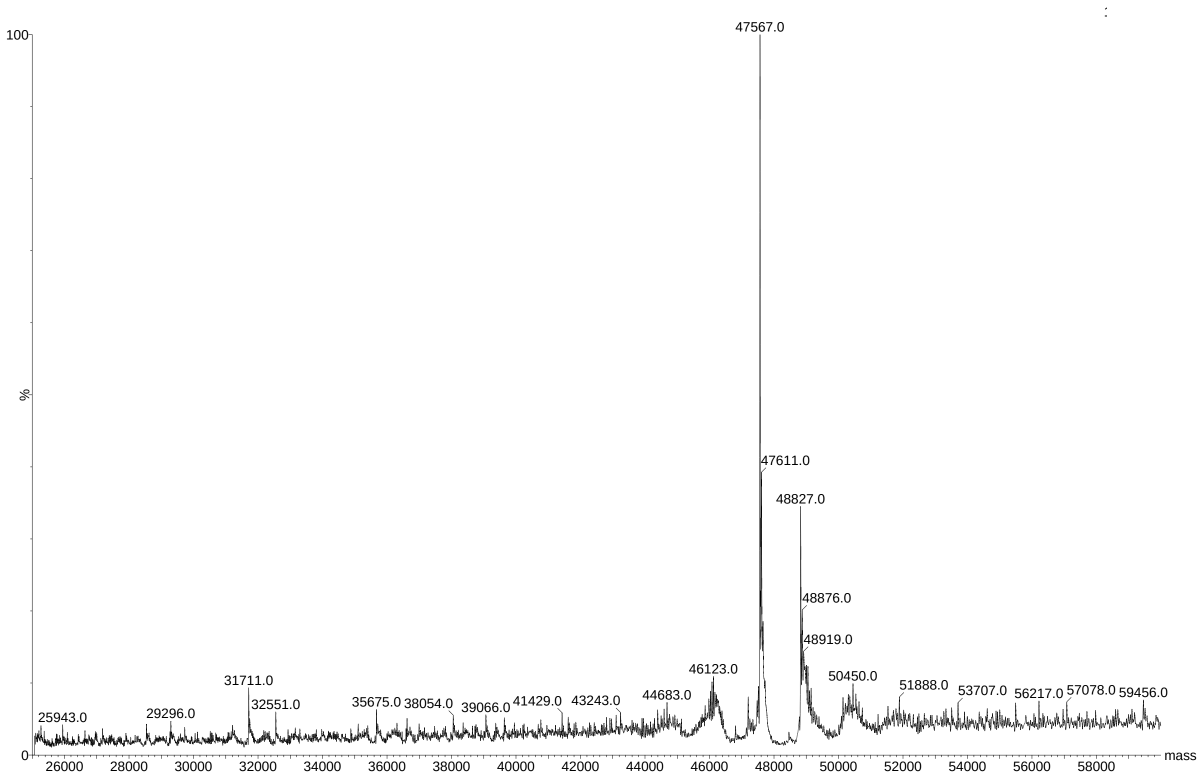

Figure S7 ESI-deconvoluted mass spectrum of the reaction between trastuzumab N_3-WT and DBCO-PEG$_4$-DFO, to yield a mixture containing unmodified WT Fab and DFO-WT.

A

Matched peptides shown in ***bold red***.

```
  1 EVQLVESGGG LVQPGGSLRL SCAASGFNIK DTYIHWVRQA PGKGLEWVAR
 51 IYPTNGYTRY ADSVKGRFTI SADTSKNTAY LQMNSLRAED TAVYYCSRWG
101 GDGFYAMDYW GQGTLVTVSS ASTKGPSVFP LAPSSKSTSG GTAALGCLVK
151 DYFPEPVTVS NSGALTSGVH TFPAVLQSSG LYSLSSVVTV PSSSLGTQTY
201 ICNVNHKPSN TKVDKKVEPK SCDKTHT
```

(Matched in bold red: 1–38 EVQLVESGGG LVQPGGSLRL SCAASGFNIK DTYIHWVR; GLEWVAR; 51–136 IYPTNGYTRY … LAPSSK.)

B

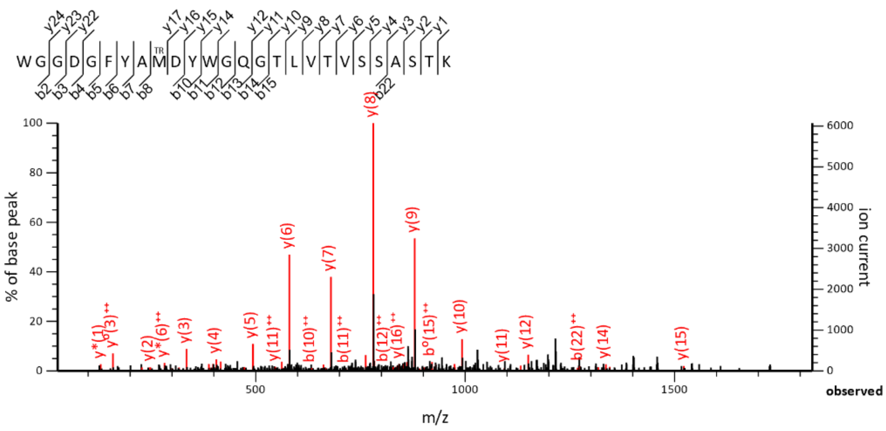


```
Monoisotopic mass of neutral peptide Mr(calc): 2950.3345
Variable modifications:
  M9 : Trastuzmab minor mod (M)
Ions Score: 30  Expect: 0.0033   (help)
```

#	b	b^{++}	b^{*}	b^{*++}	b^{0}	b^{0++}	Seq.	y	y^{++}	y^{*}	y^{*++}	y^{0}	y^{0++}	#
1	**187.0866**	94.0469					W							26
2	**244.1081**	122.5577					G	2765.2624	1383.1348	2748.2359	**1374.6216**	2747.2518	1374.1296	25
3	**301.1295**	151.0684					G	2708.2409	1354.6241	2691.2144	1346.1108	2690.2304	1345.6188	24
4	**416.1565**	208.5819			**398.1459**	199.5766	D	2651.2195	1326.1134	2634.1929	**1317.6001**	2633.2089	1317.1081	23
5	**473.1779**	237.0926			455.1674	**228.0873**	G	2536.1925	1268.5999	2519.1660	1260.0866	2518.1820	1259.5946	22
6	**620.2463**	310.6268			602.2358	301.6215	F	2479.1711	1240.0892	2462.1445	1231.5759	2461.1605	1231.0839	21
7	**783.3097**	392.1585			765.2991	**383.1532**	Y	2332.1027	1166.5550	2315.0761	1158.0417	2314.0921	1157.5497	20
8	**854.3468**	427.6770			836.3362	418.6717	A	2169.0393	1085.0233	2152.0128	1076.5100	2151.0288	1076.0180	19
9	1152.4680	**576.7376**			1134.4574	567.7323	M	2098.0022	1049.5047	2080.9757	1040.9915	2079.9917	1040.4995	18
10	1267.4949	634.2511			1249.4844	**625.2458**	D	1799.8810	900.4441	1782.8545	891.9309	1781.8705	891.4389	17
11	1430.5582	715.7828			1412.5477	**706.7775**	Y	1684.8541	842.9307	1667.8275	834.4174	1666.8435	833.9254	16
12	1616.6376	**808.8224**			1598.6270	**799.8171**	W	**1521.7907**	761.3990	1504.7642	752.8857	1503.7802	**752.3937**	15
13	1673.6590	**837.3332**			1655.6485	828.3279	G	**1335.7114**	668.3594	1318.6849	659.8461	**1317.7009**	659.3541	14
14	1801.7176	901.3624	1784.6911	892.8492	1783.7070	892.3572	Q	1278.6900	639.8486	1261.6634	631.3353	1260.6794	630.8433	13
15	1858.7391	**929.8732**	1841.7125	921.3599	1840.7285	**920.8679**	G	**1150.6314**	575.8193	**1133.6048**	**567.3061**	**1132.6208**	566.8141	12
16	1959.7867	980.3970	1942.7602	971.8837	1941.7762	971.3917	T	**1093.6099**	547.3086	1076.5834	538.7953	**1075.5994**	538.3033	11
17	2072.8708	1036.9390	2055.8443	1028.4258	2054.8602	1027.9338	L	**992.5623**	496.7848	975.5357	**488.2715**	**974.5517**	487.7795	10
18	2171.9392	1086.4733	2154.9127	1077.9600	2153.9287	1077.4680	V	**879.4782**	**440.2427**	**862.4516**	431.7295	**861.4676**	431.2374	9
19	2272.9869	1136.9971	2255.9604	1128.4838	2254.9763	1127.9918	T	**780.4098**	390.7085	**763.3832**	**382.1953**	**762.3992**	381.7032	8
20	2372.0553	1186.5313	2355.0288	1178.0180	2354.0448	1177.5260	V	**679.3621**	**340.1847**	**662.3355**	331.6714	**661.3515**	331.1794	7
21	2459.0873	1230.0473	2442.0608	1221.5340	2441.0768	1221.0420	S	**580.2937**	290.6505	**563.2671**	**282.1372**	**562.2831**	281.6452	6
22	2546.1194	**1273.5633**	2529.0928	1265.0500	2528.1088	1264.5580	S	**493.2617**	247.1345	476.2351	238.6212	475.2511	238.1292	5
23	2617.1565	1309.0819	2600.1299	1300.5686	2599.1459	1300.0766	A	**406.2296**	203.6185	389.2031	195.1052	**388.2191**	194.6132	4
24	2704.1885	1352.5979	2687.1620	1344.0846	2686.1780	1343.5926	S	**335.1925**	168.0999	318.1660	159.5866	**317.1819**	**159.0946**	3
25	2805.2362	1403.1217	2788.2096	1394.6085	2787.2256	1394.1165	T	**248.1605**	124.5839	231.1339	116.0706	230.1499	115.5786	2
26							K	147.1128	74.0600	**130.0863**	65.5468			1

Figure S8 Peptide mapping on N_3-WT Fab using a trypsin digest, confirming modification of HC.M107. (A) Sequence coverage of the mapping experiment (B) MS/MS spectrum of peptide fragment containing M107 indicating a single modification (+167.1686 Da).

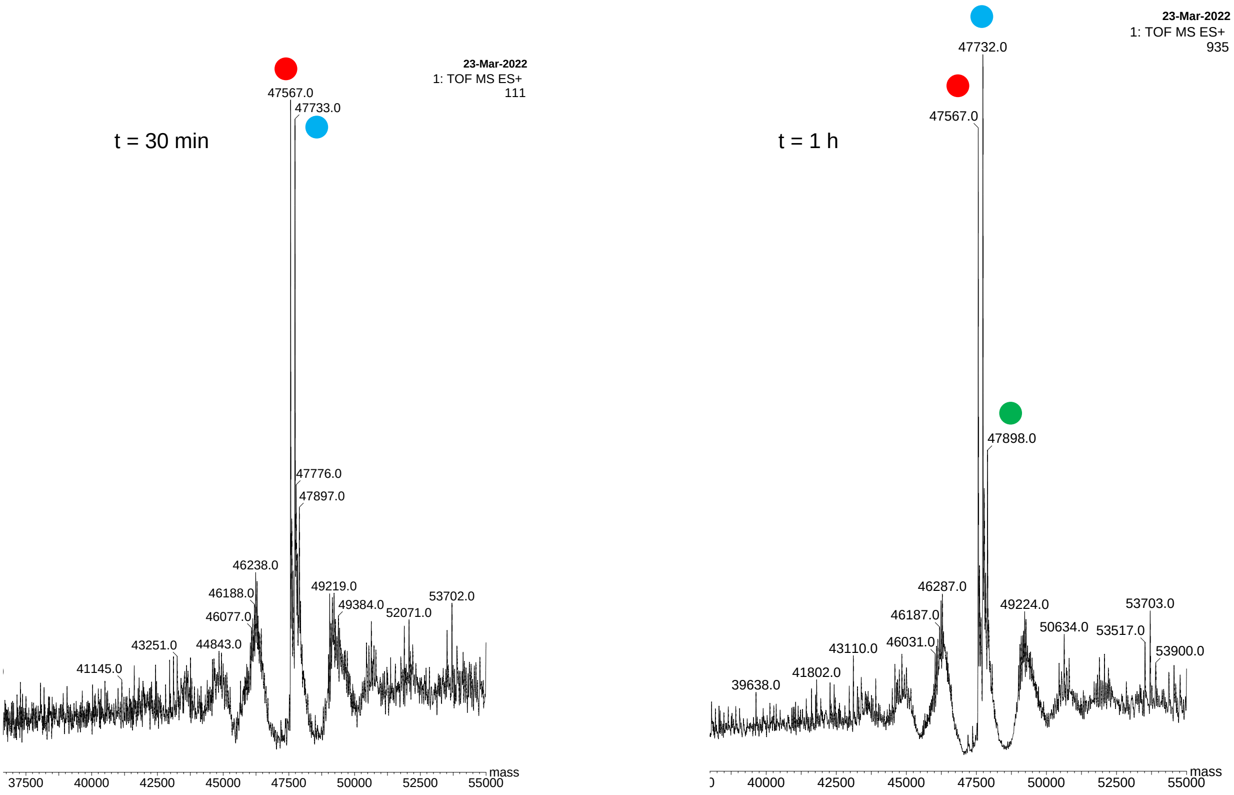

Figure S9 ESI-deconvoluted mass spectra of the reaction between trastuzumab WT Fab and 20 eq. of Ox-N_3 **1.** Signals observed at 47566 m/z (labelled red) corresponds to unmodified WT Fab and 47732 m/z (labelled blue) corresponds to singly modified WT Fab conjugate and 47898 m/z (labelled green) corresponds to dual modified WT Fab conjugate.

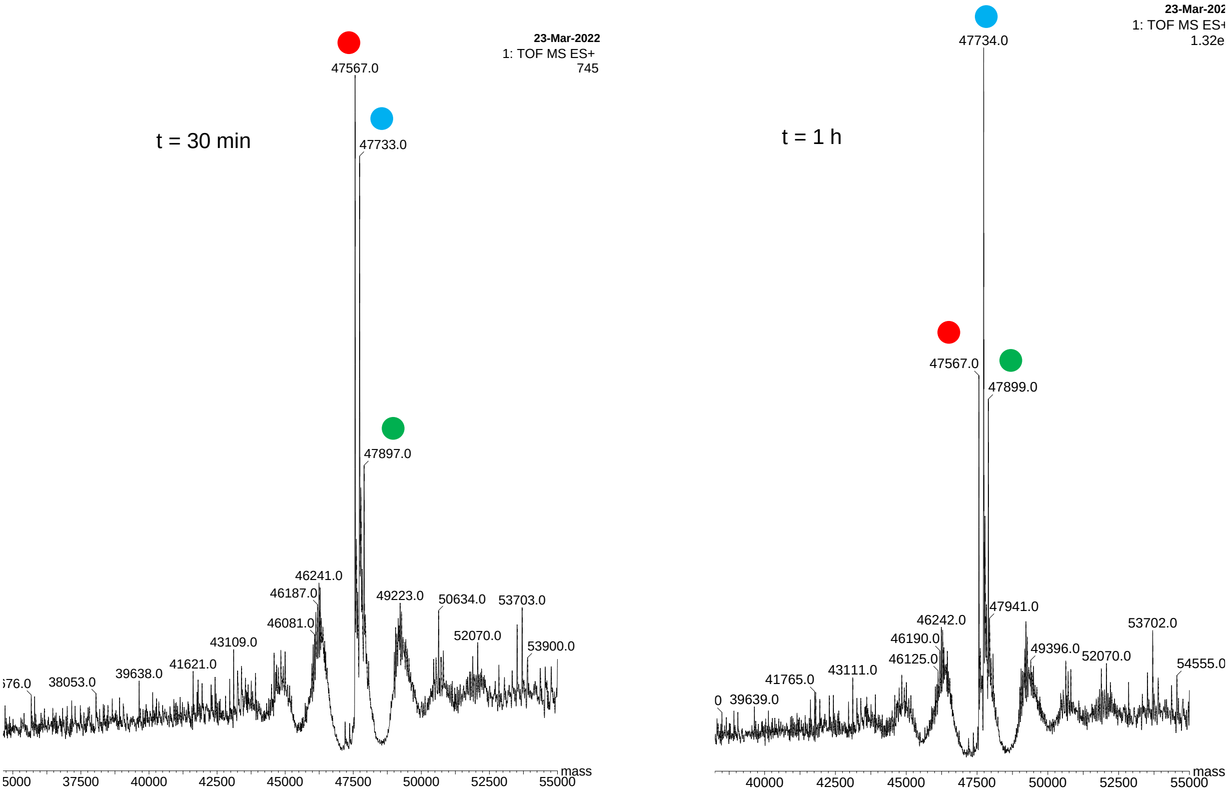

Figure S10 ESI-deconvoluted mass spectra of the reaction between trastuzumab WT Fab and 30 eq. of Ox-N_3 **1.** Signals observed at 47566 m/z (labelled red) corresponds to unmodified WT Fab and 47732 m/z (labelled blue) corresponds to singly modified WT Fab conjugate and 47898 m/z (labelled green) corresponds to dual modified WT Fab conjugate.

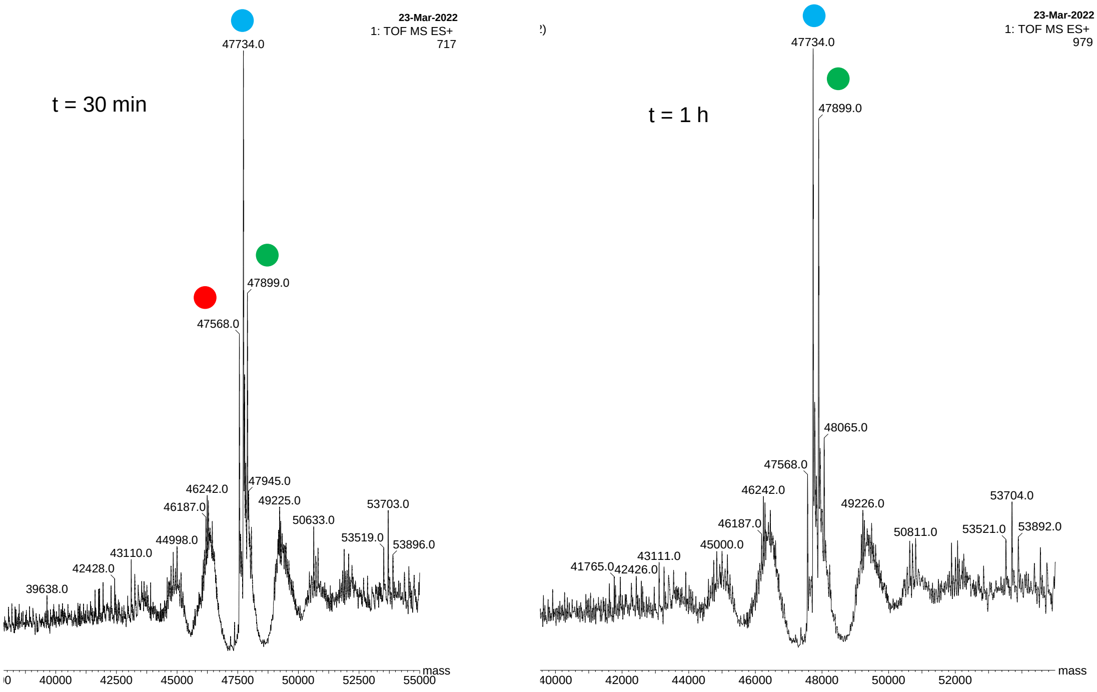

Figure S11 ESI-deconvoluted mass spectra of the reaction between trastuzumab WT Fab and 50 eq. of Ox-N_3 **1.** Signals observed at 47566 m/z (labelled red) corresponds to unmodified WT Fab and 47732 m/z (labelled blue) corresponds to singly modified WT Fab conjugate and 47898 m/z (labelled green) corresponds to dual modified WT Fab conjugate.

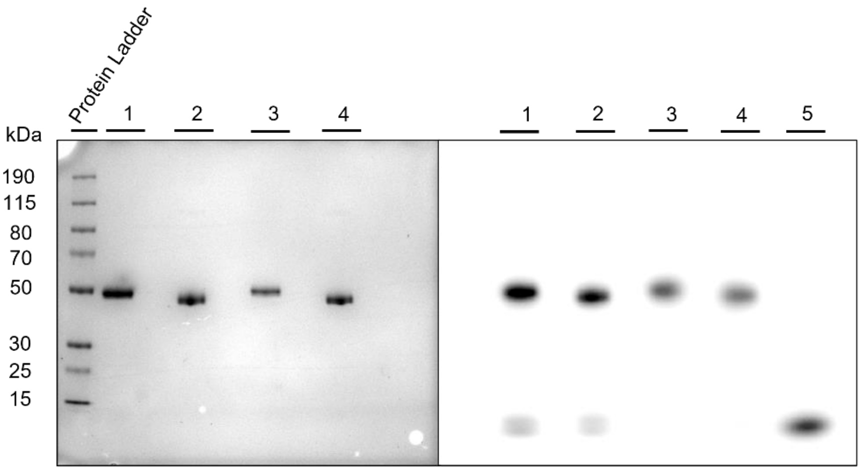

Lanes	
1	Crude [^{68}Ga]Ga-DFO-M74
2	Crude [^{68}Ga]Ga-DFO-WT
3	Zeba Spin purified [^{68}Ga]Ga-DFO-M74
4	Zeba Spin purified [^{68}Ga]Ga-DFO-WT
5	Unchelated [^{68}Ga]Ga

Figure S12 SDS PAGE analysis (Left: bright view image, Right: autoradiography) of [^{68}Ga]Ga-DFO-M74 and [^{68}Ga]Ga-DFO-WT from crude labelling reactions (1 and 2) or after Zeba spin purification (3 and 4). Unchelated [^{68}Ga]Ga migrates to bottom of SDS-gel. The radioactivity signal from the radiolabelled conjugates were coincident with the stained protein bands corresponding to the M74 and WT Fab fragments.

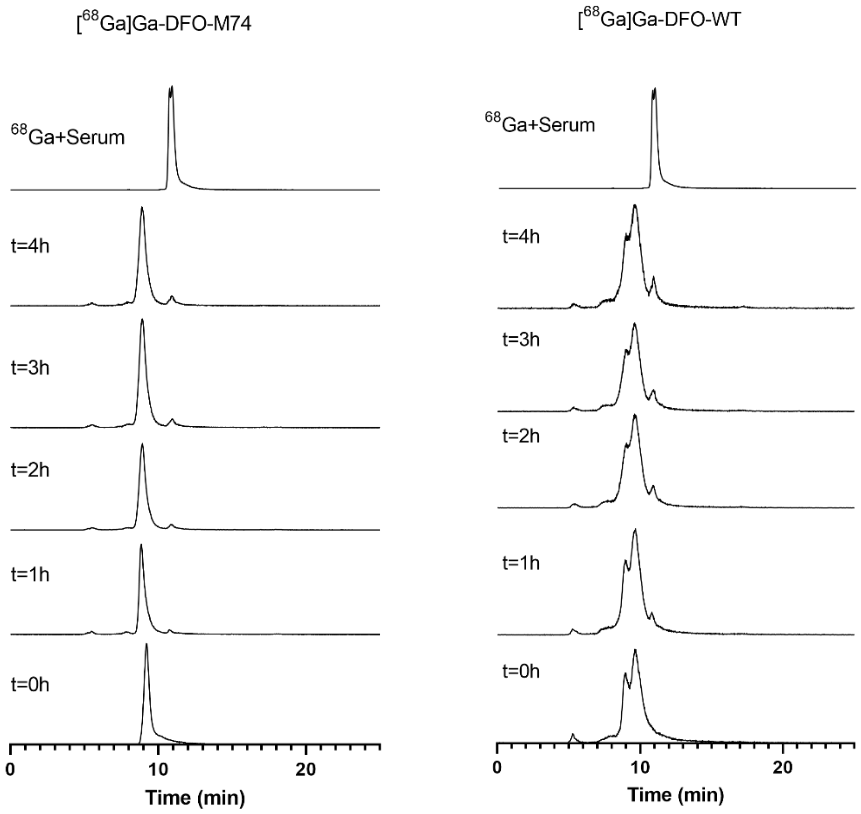

Figure S13 LEFT: SE-HPLC chromatograms of [^{68}Ga]Ga-DFO-M74 after incubation with human serum for 1, 2, 3, and 4 h at 37 ^{o}C, 95 % of conjugate remained intact after 4 h. When [^{68}Ga]Ga^{3+} was incubated with serum for 4 h at 37 ^{o}C, one signal was observed, corresponding to unreacted [^{68}Ga]Ga^{3+}. Right: SE-HPLC chromatograms of [^{68}Ga]Ga-DFO-WT after incubation with human serum for 1, 2, 3, and 4 h at 37 ^{o}C, 93 % of conjugate remained intact after 4 h.

Table S1 In vitro cell binding study of [^{68}Ga]Ga-DFO-M74 and [^{68}Ga]Ga-DFO-WT with HER2-positive HCC1954 cells, HER2-deficient cells MDA-MB-231 and under blocking conditions through addition of 200-fold unmodified Trastuzumab.

	[^{68}Ga]Ga-DFO-M74		[^{68}Ga]Ga-DFO-WT	
	Mean Uptake (% added radioactivity)	Standard Deviation	Mean Uptake (% added radioactivity)	Standard Deviation
HCC1954	14.434	3.756	8.390	2.717
HCC1954 + Blocked	0.672	0.211	0.840	0.478
MDA-MB-231	0.592	0.173	0.809	0.345
MDA-MB-231 + Blocked	0.438	0.106	0.742	0.290

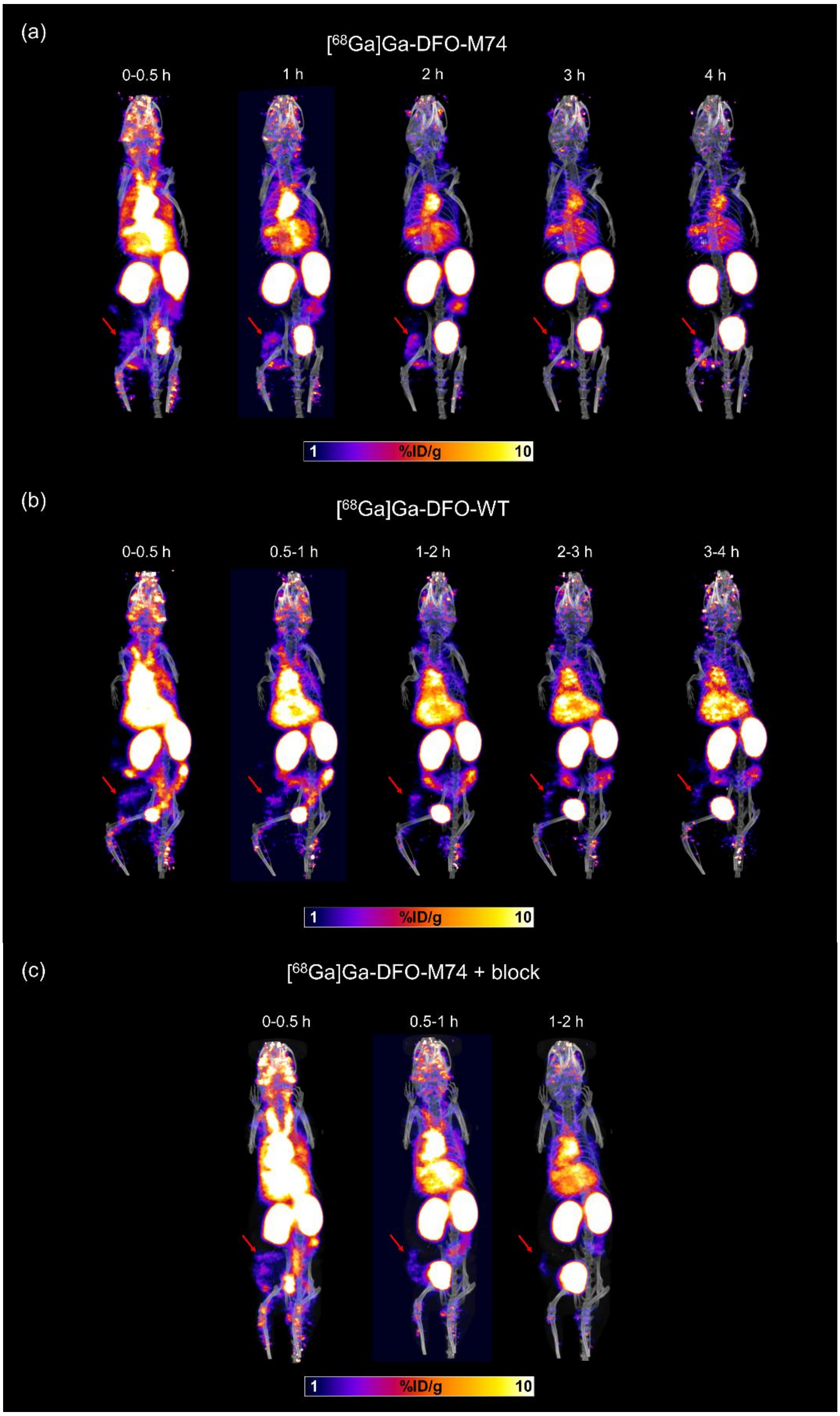

Figure S14 Maximum intensity projections (MIPs) of mice injected with (a) [^{68}Ga]Ga-DFO-M74, (b) [^{68}Ga]Ga-DFO-WT and (c) [^{68}Ga]Ga-DFO-M74 + blocking dose. Dynamic PET scans were obtained over 4 h p.i. and combined in 30 min or 1 h intervals. Red arrow indicates HCC1954 tumour sites. (n=1/bioconjugate)

Table S2 *Ex vivo* biodistribution data of [^{68}Ga]Ga-DFO-M74 and [^{68}Ga]Ga-DFO-WT in HCC1954 tumour-bearing mice at 2 h p.i. (n=3)

Ex Vivo Biodistribution Uptake (%ID/g)				
	[^{68}Ga]Ga-DFO-M74		[^{68}Ga]Ga-DFO-WT	
	Mean (n=3)	Standard Deviation	Mean (n=3)	Standard Deviation
Blood	5.192	0.782	9.415	1.137
Heart	1.940	0.091	3.408	0.654
Lungs	2.433	0.286	4.267	0.485
Liver	2.134	0.218	9.032	1.619
Spleen	2.214	0.406	10.306	2.789
Kidney	125.636	27.868	176.219	33.371
Muscle	0.218	0.033	0.306	0.082
Bone	0.497	0.100	1.756	0.384
Skin + Fur	0.689	0.130	0.880	0.107
Ovaries	2.549	0.475	4.342	0.592
Uterine horn	5.102	1.545	5.634	1.551
Tumour	1.602	0.625	1.260	0.229

Table S3 *Ex vivo* biodistribution tumour to organ ratio of [^{68}Ga]Ga-DFO-M74 and [^{68}Ga]Ga-DFO-WT in HCC1954 tumour-bearing mice at 2 h p.i. (n=3)

Tumour-to-non-targeted organ ratios				
	[^{68}Ga]Ga-DFO-M74		[^{68}Ga]Ga-DFO-WT	
	Mean (n=3)	Standard Deviation	Mean (n=3)	Standard Deviation
Blood	0.327	0.178	0.133	0.015
Heart	0.837	0.361	0.370	0.035
Lungs	0.643	0.188	0.293	0.021
Liver	0.743	0.271	0.140	0.010
Spleen	0.710	0.202	0.127	0.025
Kidney	0.013	0.006	0.010	0.000
Muscle	7.750	4.193	4.507	2.145
Bone	3.170	0.814	0.730	0.135
Skin + Fur	2.280	0.577	1.430	0.219
Ovaries	0.653	0.331	0.293	0.067
Uterine horn	0.330	0.165	0.237	0.083

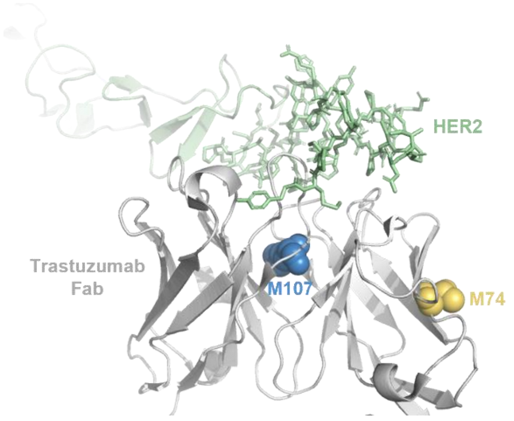

Figure S15 Crystal structure (PDB: 6BGT) of Trastuzumab Fab (grey) in complex with HER2 extracellular domain (green). HC.107 and LC.74 labelled in blue and yellow respectively. HC.107 sits in the CDR H3 antigen binding site while LC.74 is situated away from the antigen binding site. Image generated using PyMOL version 2.5.2.

References

(1) Elledge, S. K.; Tran, H. L.; Christian, A. H.; Steri, V.; Hann, B.; Dean Toste, F.; Chang, C. J.; Wells, J. A. Systematic Identification of Engineered Methionines and Oxaziridines for Efficient, Stable, and Site-Specific Antibody Bioconjugation. *Proc. Natl. Acad. Sci. U. S. A.* **2020**, *117* (11), 5733–5740.

(2) Ahn, S. H.; Vaughn, B. A.; Solis, W. A.; Lupher, M. L.; Hallam, T. J.; Boros, E. Site-Specific 89Zr- and 111In-Radiolabeling and In Vivo Evaluation of Glycan-Free Antibodies by Azide–Alkyne Cycloaddition with a Non-Natural Amino Acid. *Bioconjugate Chem.* **2020**, *31* (4), 1177–1187.

(3) Tang, Y.; Wang, J.; Scollard, D. A.; Mondal, H.; Holloway, C.; Kahn, H. J.; Reilly, R. M. Imaging of HER2/Neu-Positive BT-474 Human Breast Cancer Xenografts in Athymic Mice Using 111In-Trastuzumab (Herceptin) Fab Fragments. *Nucl. Med. Biol.* **2005**, *32* (1), 51–58.
